# Matrix-Resolved Profiling of the Prenatal Chemical Exposome Reveals Selective Placental Retention and Fetal Transfer

**DOI:** 10.1021/acs.est.6c02272

**Published:** 2026-09-24

**Authors:** Montse Marquès, Ruben Gil-Solsona, Ana González-Ruiz, Esteban Restrepo-Montes, Ana Megía, Monica Ballesteros, José L. Domingo, Ana Navas-Acien, Gary W. Miller, Adrian Covaci, Pablo Gago-Ferrero

**Affiliations:** † 203229 Institute of Environmental Assessment and Water Research (IDAEA-CSIC), Barcelona 08034, Spain; ‡ Toxicological Centre, Department of Pharmaceutical Sciences, 26660 University of Antwerp, Antwerpen 2610, Belgium; § Department of Environmental Health Sciences, 5798 Columbia University, New York 10032, NY, United States; ∥ School of Medicine, 73047 Universitat Rovira i Virgili, Laboratory of Toxicology and Environmental Health, TecnATox, School of Medicine, Reus 43201, Spain; ⊥ Institut d’Investigació Sanitària Pere Virgili (IISPV), Doctor Mallafré Guasch 4, Tarragona 43005, Spain; # PhD Program “Analytical Chemistry and Environment”, Universitat de Barcelona, Barcelona 08028, Spain; ¶ Department of Endocrinology and Nutrition, Research Unit, University Hospital of Tarragona Joan XXIII-Institut d’Investigació Sanitària Pere Virgili (IISPV), Tarragona 43005, Spain; ∇ Department of Obstetrics and Gynecology, University Hospital of Tarragona Joan XXIII Institut d’Investigació Sanitària Pere Virgili (IISPV), Tarragona 43005, Spain

**Keywords:** Maternal-fetal exposome, transplacental transfer, human biomonitoring, high-resolution mass spectrometry, pregnancy, prenatal exposure

## Abstract

Chemical exposure during human development, especially in utero, can disrupt many biological processes and increase disease vulnerability. Assessing the chemical landscape across the maternal–fetal interface is challenging. Here we address this challenge by conducting an exposome-based analysis across a suite of integrated maternal–fetal biological compartments: maternal urine, maternal serum, placental tissue, and umbilical cord serum. Using wide-scope liquid chromatography–high-resolution mass spectrometry (LC-HRMS) in 23 mother-infant dyads from the INSULIN cohort (Spain), we profiled 659 chemicals and transformation products, enabling a comprehensive characterization of prenatal chemical exposure and its compartment-specific distribution. We identified 76 unique compounds, including food additives, personal care products, pharmaceuticals, plasticizers, pesticides, flame retardants, industrial chemicals, and tobacco-related biomarkers. Matrix-resolved profiling revealed distinct compartmentalization patterns, with lipophilicity (logP) emerging as the primary physicochemical determinant of placental retention and transplacental transfer, while active transport mechanisms governed the fetal transfer of selected compounds despite properties inconsistent with passive diffusion. We report, for the first time, the transplacental passage of multiple emerging contaminants, including organophosphate esters, food-related compounds and personal care product ingredients. In contrast, several other contaminants—such as selected biomarkers of plastics, smoke, and personal care products—showed no evidence of transplacental transfer. These findings highlight the value of comprehensive, multi-matrix exposome profiling for assessing prenatal chemical exposures and provide a scalable framework for exposome-wide association studies in pregnancy cohorts and beyond.

## Graphical abstract

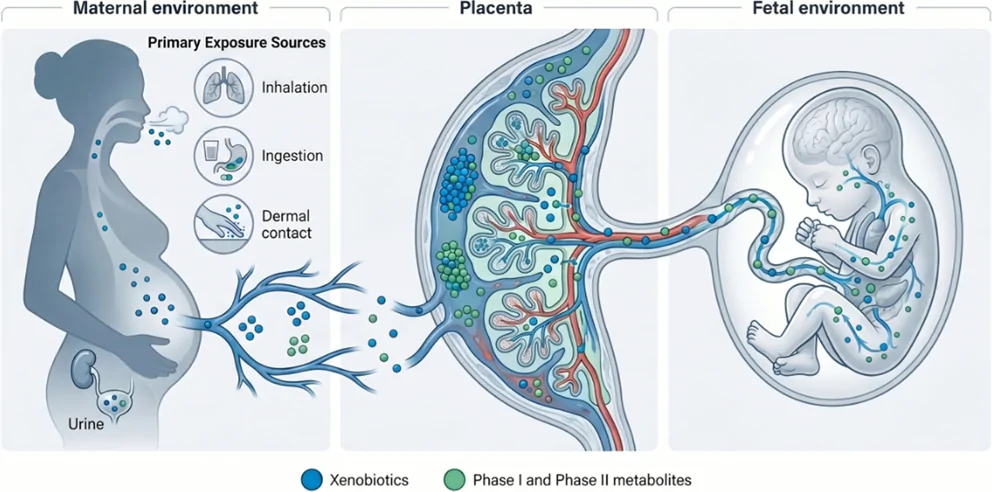

## 1 Introduction

Human exposure to a rapidly expanding array of environmental, occupational, and consumer-related xenobiotics is increasing worldwide. Industrialization, technological advances, and shifting lifestyles have immersed individuals in a complex chemical landscape originating from diverse sources. While the long-term health implications of many of these exposures remain uncertain, growing evidence suggests they may contribute to chronic disease risk.^1^ Understanding how this evolving chemical environment interacts with human biology requires integrative, systems-level approaches that capture both exposure and response dynamics—a need increasingly recognized across biomedical research.^2^

Pregnancy represents a critical window of susceptibility to environmental insults.^3^ The profound hormonal, metabolic and immunological changes during gestation make women especially vulnerable. This vulnerability can disrupt maternal–fetal homeostasis and increase the risk of adverse pregnancy outcomes, including preterm birth and low birth weight,^4,5^ and also long-term health outcomes during adulthood due to metabolic reprogramming.^6,7^ The rising global burden of such outcomes mirrors the rapid increase in chemical production and exposure, underscoring the urgency of addressing this issue. Emerging evidence continues to identify newly EDCs and other toxic chemicals, reinforcing the urgency of proactive surveillance and intervention.^8^

Despite the recognized risks posed by chemical mixtures, current human biomonitoring (HBM) approaches remain constrained by methodological and conceptual limitations. Most large-scale studies rely on targeted analytical panels focused on a narrow subset of chemical classes,^9^ or analyze biospecimens in isolation,^10,11^ limiting a systems-level understanding of internal chemical diversity. This compartmentalized approach constrains our ability to map the full breadth of xenobiotic exposure—i.e., whether compounds persist in circulation, bioaccumulate in tissue, or traverse biological barriers such as the placenta. Recent advances in high-resolution mass spectrometry (HRMS) offer unprecedented opportunities to map the internal chemical exposome at scale.^12,13^ When applied across multiple biological matrices with distinct anatomical and physiological roles, HRMS enables reconstruction of intra-individual chemical partitioning and inter-compartmental dynamics, including placental retention and transplacental transfer, across a broad chemical space that extends well beyond what pre-defined targeted panels can capture. Yet, this capacity remains largely underutilized in pregnancy research. Most existing studies do not simultaneously sample maternal, placental, and fetal compartments, nor do they capture real-world chemical diversity using broad-spectrum analytical methods. Recent exposome-wide and multi-omics initiatives have demonstrated the value of integrative strategies in uncovering early-life molecular signatures of chemical exposures.^14,15^

In this study, we present a systems-level approach to characterize the maternal–fetal chemical exposome using wide-scope LC-HRMS. We collected maternal urine, maternal serum, placenta, and umbilical cord serum, representing an integrated set of maternal–fetal biological matrices that allow interrogation of chemical transport dynamics across the maternal–fetal interface. These samples were analyzed from 23 participant dyads in the INSULIN cohort (Catalonia, Spain). By integrating data across these physiologically distinct compartments we aim to (1) comprehensively characterize the chemical exposome during late pregnancy, (2) investigate matrix-specific patterns of chemical distribution within the maternal–fetal system, and (3) identify physicochemical properties associated with placental retention and transplacental transfer for different chemicals. This work provides a high-resolution view of internal chemical partitioning during pregnancy and establishes a scalable framework for future exposome-wide studies targeting vulnerable developmental periods during pregnancy and early life.

## 2 Methods

### 2.1 Sample Collection

This pilot study leveraged samples from 23 participants of INSULIN population-based cohort study (Figure 1A). Between March 2021 and January 2022, pregnant women attending the 12 week appointment at Hospital Universitari de Tarragona Joan XXIII (Tarragona, Catalonia, Spain) were invited to join the INSULIN cohort. Exclusion criteria were pre-existing type 1 or type 2 diabetes, and major neonatal congenital anomalies. Participants were given an information sheet and signed informed consent forms prior to enrollment. At recruitment, participants’ pre-pregnancy weight and age were recorded, and gestational age was confirmed by routine ultrasonography. Each participant then self-collected a spot urine sample in a sterile polypropylene container. At 30 weeks of gestation, socio-demographic information—including educational level, occupation, frequency of household pesticide use, and use of personal care products (e.g., makeup, soap, and hair care items)—was obtained using a structured, self-administered questionnaire specifically developed for this study. On the day of delivery, upon arrival at the hospital, participants provided a spot urine sample in a sterile polypropylene container, ensuring temporal alignment with the biological matrices collected at delivery. In addition, 10 mL of maternal blood was drawn from the antecubital vein using BD Vacutainer Rapid Serum Tubes. After delivery, the gestational age was documented, and a trained midwife collected 10 mL of cord blood with BD Vacutainer Rapid Serum Tubes, and pooled samples from each quadrant of placenta (1 cm^3^) were taken, rinsed in cold 1× PBS and frozen in liquid nitrogen. Samples were stored at −80 °C in the INSULIN collection of biological samples located at the biobank of Pere Virgili Health Research Institute. INSULIN study complied with all ethical regulations and was approved by the ethics committee of Pere Virgili Health Research Institute (Ref. 225/2020).

**Figure 1:**
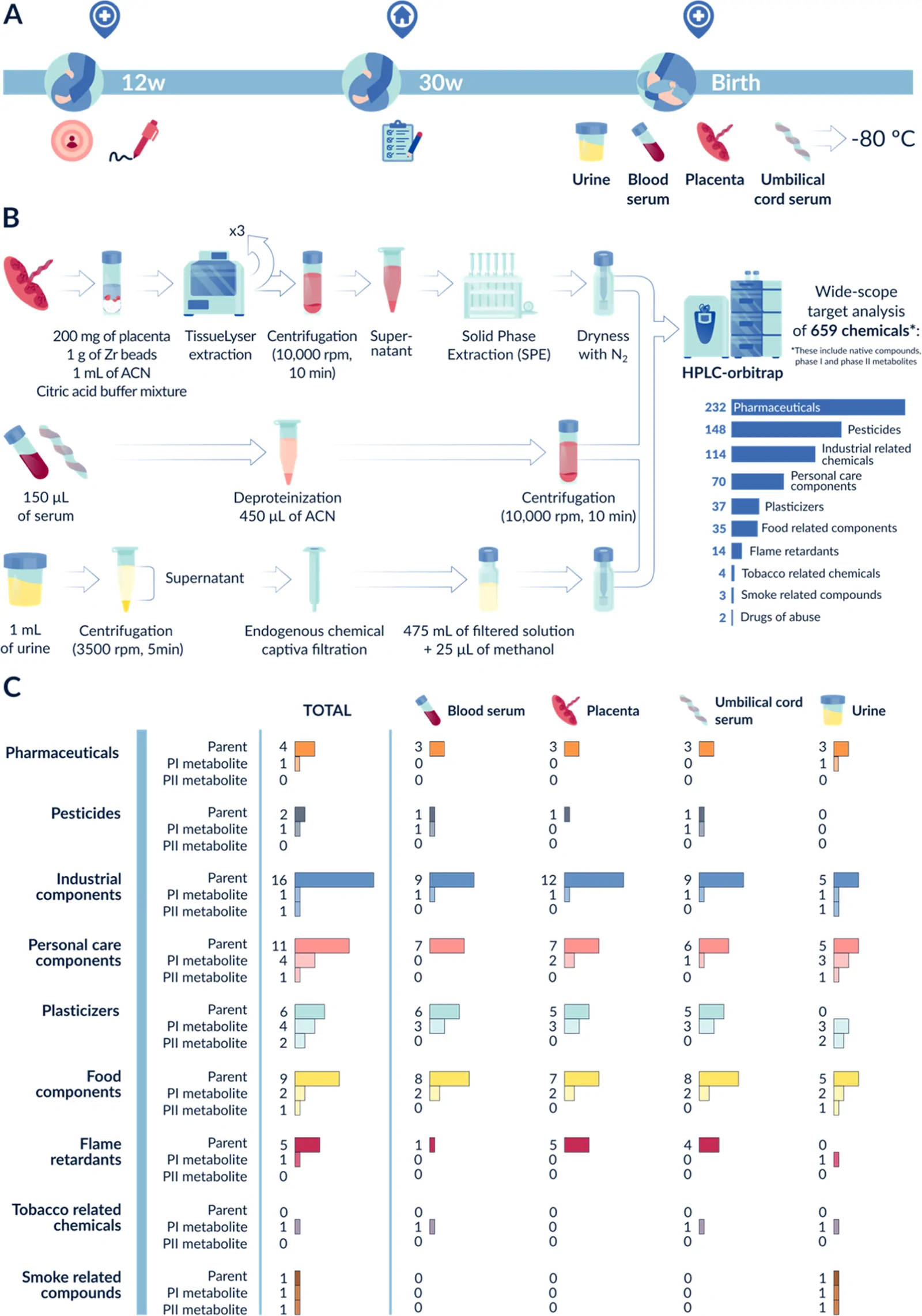
**Study design, analytical workflow, and chemical coverage across maternal and fetal compartments**. (A) overview of participant recruitment, dietary and lifestyle data collection, and biospecimen sampling within the INSULIN longitudinal cohort. (B) Schematic of the generic sample preparation and analysis workflow. The accompanying histogram displays the number of chemicals per chemical family included in the wide-scope target library. (C) Distribution of detected parent compounds, phase I (PI) metabolites, and phase II (PII) metabolites by chemical class and biospecimen (maternal serum, placenta, umbilical cord serum and urine).

### 2.2 Sample Preparation

The sample preparation and analytical workflow are summarized in Figure 1B; three matrix-specific extraction protocols were applied, one for serum (maternal and cord), placental tissue, and urine. All solvents, reagents, standards, and SPE materials were of LC–MS grade or higher purity; a full list with suppliers is provided in the Supporting Information (Section S1).

Sample extraction for urine was adapted from a previously published protocol.^16^ The placental tissue extraction method was based on a protocol originally developed for brain tissue^17^ and subsequently validated for placenta with acceptable performance. The serum extraction method was developed and validated in-house. All samples were spiked prior to extraction with clothianidin-*d*_3_, as a surrogate standard to monitor extraction performance, reaching a concentration of 50 ng/mL in serum and urine and ∼50 ng/g in placenta tissue; spiking volumes are detailed in Section S2.

Maternal and cord serum were prepared by acetonitrile protein precipitation. Briefly, 450 μL of acetonitrile was added to 150 μL serum, and the samples were shaken to ensure homogeneous protein precipitation and centrifuged (10,000 rpm, 10 min). The resulting supernatant was transferred directly to chromatographic vials. This in-house method, validated for both serum matrices (Table S1), deliberately omitted evaporation and reconstitution, accepting an ∼4-fold dilution, to avoid concentrating phospholipids and increasing matrix effects.

Placental tissue (∼200 mg) was first extracted with a tissuelyzer using zirconium oxide beads and citrate buffer/acetonitrile solution (1:1, v/v). The combined extracts were then cleaned up and concentrated by solid-phase extraction (SPE) on home-made cartridges.^18^ Briefly, the pooled extract was diluted in Milli-Q water to below 5% acetonitrile, loaded onto the cartridges, eluted, evaporated under nitrogen, and reconstituted to a 1× concentration factor (selected over 2× and 5× during optimization). This method is validated (Table S2) and full extraction and clean-up conditions are provided in Section S2.

Urine (1 mL) was subjected to a minimal preparation comprising centrifugation and direct filtration through a Captiva filtration cartridge, following a previously published protocol.^16^ Urinary creatinine was determined in parallel^19^ to account for urine dilution. Full conditions are provided in Section S2.

### 2.3 LC-HRMS Analysis

Instrumental analysis was performed using an Acquity UHPLC system (Waters) coupled to a Q-Exactive Orbitrap mass spectrometer (Thermo Fisher Scientific). Chromatographic separation was achieved on a Cortecs C18 column (2.1 × 100 mm, 2.7 μm) with a matching precolumn, with electrospray ionization (ESI) operated at 3000 V (positive mode) and 2800 V (negative mode). Key parameters were: capillary temperature 350 °C, sheath gas 40 a.u., auxiliary gas 10 a.u., probe heater 350 °C, and lens RF level 60. Data were acquired in data-independent acquisition (DIA) mode with full scans at low (4 eV) and high (25 eV) collision energy, covering 67–1000 at a resolution of 60,000. Full details of the LC method, including mobile phase composition, gradient programme, run time, injection volume, and column temperature, are reported in a previously published protocol and can be consulted elsewhere.^18^

### 2.4 Data Evaluation and Quantification

Quantification relied on matrix-matched calibration curves prepared for each matrix from pooled QC samples (equal volumes of all deproteinized serum, all extracted urine, and aliquots of a single extracted placenta sample), together with a parallel solvent calibration curve. These curves enabled absolute quantification for compounds with available analytical standards (80 chemicals, Table S3) and supported a validated matrix-specific semiquantification model for compounds lacking individual standards, incorporating matrix-specific ionisation effects to reduce bias in cross-matrix comparisons. Full calibration construction and the model’s development and validation are detailed in Section S3 and in a dedicated methods manuscript in preparation; method performance data are provided in Tables S1 and S2.

Target compounds were assigned Level 1 confidence (Schymanski scale^20^) on the basis on (i) precursor ion exact mass within ±0.002 Da, (ii) retention time within ±0.15 min of database values, and (iii) presence of the most abundant fragment in the high-energy spectrum, with additional confirmation by correct isotopic pattern and secondary diagnostic fragments. A compound was reported as a positive finding only when its calculated concentration exceeded the threshold defined in the QA/QC section.

A suspect screening workflow was then applied to detect phase II metabolites (glucuronide conjugates) across all matrices based on (i) the exact mass of the conjugated metabolite in the low-energy scan, (ii) the precursor mass of the parent compound in the high-energy scan (neutral loss of the conjugate moiety), and (iii) a slightly earlier retention time than the parent. Tentative identifications were confirmed by fragment ions matching the corresponding to the unmetabolized compound. These metabolites were assigned Level 2b confidence.^20^

### 2.5 QA/QC

Instrumental performance was assured by calibrating the Orbitrap mass spectrometer for mass accuracy in both ionization modes at the start of each sequence (manufacturer’s guidelines), injecting calibration standards at the beginning and end of each batch, and including a QC standard (50 ng/mL) at regular intervals to track signal stability and drift. Five blanks (Milli-Q water) were processed, stored and handled identically to biological samples (in the same cryovials used for sample storage) and solvent blanks were carried though the full extraction procedure and injected at the start and end of each batch. Milli-Q water served as the blank for all four matrices because certified analyte-free placental tissue and cord serum are not commercially available and background-free status cannot be guaranteed for available serum or urine blanks (full rationale and contamination-control measures in Section S4). Concentrations of compounds detected in blanks are reported in Table S4.

Recovery, matrix effects, reproducibility (*n* = 3 at two concentration levels) for the serum and placenta levels are provided in Tables S1 and S2, respectively; urine validation has been reported previously.^16^ Mean recoveries were within 60–130% for 86%, 70%, and 75% of validated compounds in serum, placenta, and urine, respectively, and inter-matrix recovery ratios indicated that differential recovery across matrices is unlikely to bias the reported distribution patterns (Section S4).

The statistical nature of HRMS signal processing makes signal-to-noise ratios unreliable for defining limits of detection (LODs). Hence, LODs were derived per matrix in two ways: experimentally for compounds included in the matrix-matched calibration curves (lowest reliably detectable calibration point when absent from blanks, or mean blank concentration +1 SD when present), and via the matrix-specific semiquantification model for compounds not included in the curves (Table S5; model-based estimates for screened-but-undetected compounds in Table S6). A compound was reported as a positive finding only when its concentration exceeded the limit of quantitation (LOQ), defined as the mean blank concentration plus three standard deviations for compounds detected in blanks; for compounds absent from blanks, LOD and LOQ were treated as equivalent.

### 2.6 Data Analysis

Data were processed in RStudio (v2024.12.1) and Python (v3.12.3); full package details are listed in Section S3. Descriptive statistics summarised detection frequencies and concentration ranges across matrices, retaining only chemicals detected in ≥20% of samples in at least one matrix for comparative analyses. Concentrations were expressed as ng/mL for serum (maternal and cord), ng/g for placental tissue, and μg/g creatinine for urine, and log_10_-transformed for visualisation. Non-detected values were imputed as LOD/2.

The placental retention ratio was defined as the placental tissue/maternal serum concentration ratio, and the transplacental transfer ratio as the cord serum/placental tissue ratio. These complementary ratios were defined to resolve maternal–fetal transfer into its sequential steps—partitioning from maternal serum into the placenta (retention) and from the placenta to the fetal circulation (transfer)—rather than as a single cord-to-maternal ratio. Because this compartment-resolved transfer ratio differs from the conventional cord/maternal-serum ratio and places placental retention in its denominator, a high value may reflect low placental accumulation as well as efficient fetal delivery; ratios were therefore interpreted as directional indicators of compartmental partitioning rather than absolute transfer efficiencies. Both placental retention and transplacental transfer ratios were computed only for chemicals detected in ≥20% of the relevant paired biospecimens.

Spearman correlation coefficients (ρ) assessed intra-matrix associations (compound pairs within each matrix) and inter-matrix associations (compound pairs co-detected across paired matrices), restricting both to compounds with a true detection frequency ≥70% per matrix; true detections were distinguished from LOD/2 imputations by repeated identical values within each compound–matrix combination. Spearman correlations were also used to relate four physicochemical descriptors: logP, molecular weight, molar refractivity, and van der Waals volume, selected for their mechanistic relevance to membrane partitioning and transplacental transport, to placental retention and transplacental transfer. All *p*-values were corrected with the Benjamini–Hochberg false discovery rate (BH-FDR) procedure, and significance was set at *p* < 0.05.

## 3 Results

In Table 1, we present the characteristics of our study population. The mean (SD) age of pregnant women was 33 (7) years, and they delivered after a mean of 39.2 (1.6) weeks of gestation, with a pre-pregnancy body mass index (BMI) of 25.7 (6.2) kg/m^2^. Most participants were born in Spain or Latin America, held a bachelor’s degree or higher, and worked in the services sector. Common exposure-related habits included daily use of soap and make-up products, weekly use of hair products, and occasional use of domestic pesticides.

**Table 1:** Characteristics of the Study Population^e^

	total (*N* = 23)
**maternal age**,^a^ years	33 (7)
**country of birth** ^b^	
Spain	8 (32%)
Latin America	8 (32%)
Asia	1 (4%)
**BMI** ^a^, kg/m^2^	25.7 (6.2)
**gestational age** ^a^, weeks	39.2 (1.6)
**educational level** ^c^	
high school or less	3 (12.0%)
vocational training	5 (20.0%)
bachelor degree or more	11 (44%)
**profession** ^c^	
industry	6 (24%)
services	8 (32%)
education	1 (4%)
healthcare	4 (16%)
**use of domestic pesticide** ^c^	
daily	1 (4%)
monthly	1 (4%)
annually	9 (36%)
almost never/never	8 (32%)
**use of make-up** ^d^	
daily	6 (24%)
weekly	6 (24%)
monthly	2 (8%)
annually	4 (16%)
**use of soap** ^d^	
daily	15 (60%)
weekly	2 (8%)
monthly	1 (4%)
**use of hair products** ^d^	
daily	5 (20%)
weekly	11 (44%)
annually	2 (8%)

a Data available for the 23 participants.

b Data available for 17 participants.

c Data available for 19 participants.

d Data available for 18 participants.

e Continuous variables are presented as mean (SD). Categorical variables are presented as *n* (%). BMI: body mass index.

### 3.1 Occurrence of Chemicals Across Biological Matrices

Overall, our analytical pipeline allowed us to identify 76 distinct compounds, including native organic chemicals and their phase I metabolites (products of oxidation, reduction, or hydrolysis reactions that increase polarity) and phase II metabolites (conjugation products, such as glucuronides and sulfates, that further increase water solubility and facilitate excretion) (Figure 1C). These included food-related chemicals (9 parents, 2 phase I and 1 phase II metabolites); pharmaceuticals (4 parents and 1 phase I); personal care product ingredients (11 parents, 4 phase I and 1 phase II); plastic additives (6 parents, 4 phase I and 2 phase II); pesticides (2 parents, 1 phase I), flame retardants (5 parents and 1 phase I), industrial chemicals (16 parents, 1 phase I and 1 phase II), along with smoke-related compounds (1 parent, 1 phase I and 1 phase II) and 1 tobacco biomarker (phase I). Of the 76 compounds detected, the vast majority were identified at Level 1 confidence according to the Schymanski classification scale,^20^ based on matching of accurate mass, chromatographic retention time, and MS/MS fragmentation spectrum against authentic reference standards. A small number of phase II metabolites—specifically glucuronide conjugates—for which no authentic reference standard was available were classified at Level 2b, based on accurate mass and predicted fragmentation patterns. Identification confidence levels for all 76 compounds are provided in Table S7.

Figure 2 showcases the detection frequencies and concentrations of exposure biomarkers in maternal serum, placenta, umbilical cord serum, and urine (threshold of 20% of detection frequency in at least one biospecimen). Detailed occurrence of each compound per biospecimen is included in Supporting Information (Table S7). In maternal serum, chemicals detected in all participants included food components such as caffeine and its metabolites theobromine and theophylline (also used clinically to treat respiratory disorders, though here most likely reflecting caffeine metabolism), sorbitol, indole-3-acetic acid, piperine, and oxindole. The pesticide by-product 3,5-dibromo-4-hydroxybenzoic acid (biomarker of exposure to bromoxynil), industrial chemicals, including benzothiazole and its metabolite 2-hydroxybenzothiazole, perfluorinated compounds (PFOA, PFOS, and PFHxS), and phthalate metabolites (mono-2-ethylhexyl phthalate and mono-isobutyl phthalate), also exhibited 100% detection frequency. Lidocaine was detected in most participants, consistent with epidural anesthesia. Less frequently detected compounds included saccharin, the pesticide bromoxynil, personal care components such as 3,4-dihydroxybenzoic acid, *N*,*N*-diethyl-*meta*-toluamide (DEET) and ethylparaben, and bisphenols (BPS and BPAF), *N*,*N*-diphenylguanidine, lauryl diethanolamide and cotinine. Besides the drugs administered during labor (i.e.: lidocaine, acetaminophen and ampicillin), food components were those occurring in the highest range of concentrations in maternal serum (caffeine: 57.8–4000 ng/mL; sorbitol: 9.4–55 ng/mL; piperine: 2.7–130 ng/mL; theobromine: 0.63–120 ng/mL), although the occurrence of tire additives such as benzothiazole (11–36 ng/mL) and its metabolite 2-hydroxybenzothiazole (1.8–7.6 ng/mL), and other industrial chemicals like erucamide (nd-130 ng/mL), along with mono-isobutyl phthalate (0.23–23 ng/mL) and mono-2-ethylhexyl phthalate (0.89–55 ng/mL), and the personal care additive 3,4-dihydroxybenzoic acid (nd-380 ng/mL), should also be noted.

**Figure 2:**
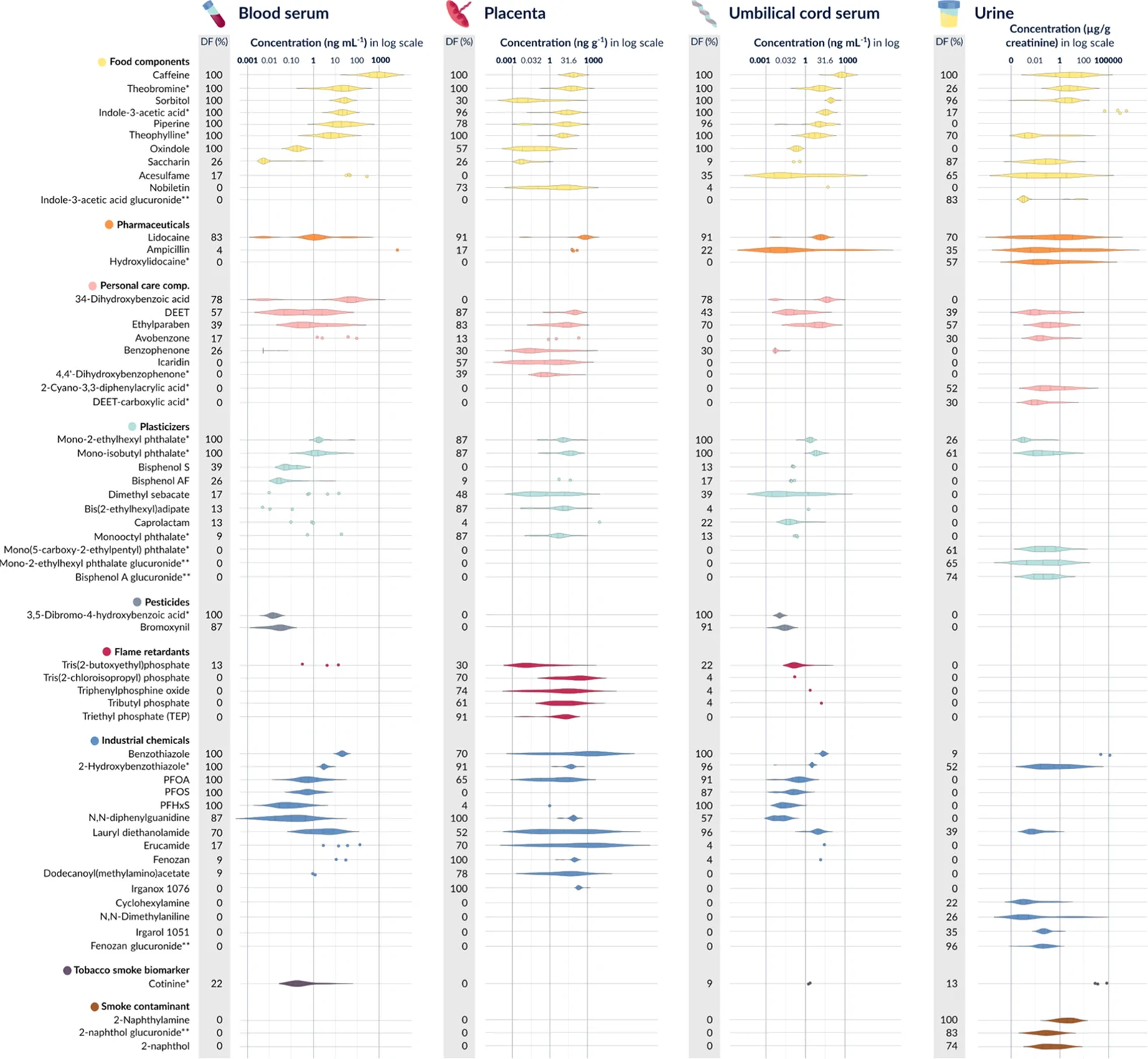
**Distribution of chemicals detected in pregnant women**. Violin plots represent the distribution of compound concentrations detected in maternal serum, placental tissue, umbilical cord serum and maternal urine. Vertical bars within each plot indicate the first quartile, the median, and the third quartile. For visualization purposes, concentrations below the limit of quantitation (LOQ) were imputed as half the LOQ. Detection frequencies (DF) are shown as numeric values (percentage) with grey backgrounds. Compounds with detection frequencies below 20 % are displayed as individual dots instead than violins to account for limited detection and avoid misleading distribution shapes. Chemicals not reaching 20 % of detection in any matrix are excluded from the figure; their concentrations and detection frequencies are reported in the Supporting Information (Table S7). * and ** refer to phase I and phase II metabolites, respectively.

Placental tissue revealed the widespread presence of food components such as caffeine, theobromine and theophylline, and industrial chemicals, such as the tire additive *N*,*N*-diphenylguanidine, the phenolic antioxidant Irganox 1076 and fenozan, a biotransformation product of various synthetic antioxidants. Other widely distributed compounds included dietary constituents (indole-3-acetic acid, piperine, nobiletin and oxindole), anesthetics (lidocaine), plasticizers (phthalate metabolites and bis­(2-ethylhexyl)­adipate) and personal care components including DEET, ethylparaben, icaridin, 4,4′-dihydroxybenzophenone, and benzophenone. Additionally, benzothiazole and its metabolite 2-hydroxybenzothiazole, erucamide, dodecanoyl­(methylamino)­acetate, lauryl diethanolamide, PFOA, and the organophosphate flame retardants, including tris­(2-chloroisopropyl) phosphate, triphenylphosphine oxide, tributyl phosphate, triethyl phosphate were also widely detected. In addition to being among the compounds most frequently detected in placenta, lidocaine (nd-3100 ng/g), benzothiazole (nd-3400 ng/g), erucamide (nd-79,000 ng/g), irganox 1076 (140–1100 ng/g), fenozan (4.0–200 ng/g), dodecanoyl­(methylamino)­acetate (nd-1700 ng/g) and tris­(2-chloroisopropyl) phosphate (nd-1200 ng/g), were the compounds with the highest concentration ranges. In turn, chemicals such as caprolactam (nd-8900 ng/g) and lauryl diethanolamide (nd-23,000 ng/g) occurred less frequently, but at remarkable levels.

Supporting Information

The widespread presence of chemicals in umbilical cord serum reflected a partially overlapping chemical profile with maternal serum findings, with consistent detection of dietary components such as caffeine, theobromine, theophylline, sorbitol, indole-3-acetic acid, oxindole, as well as phthalate metabolites (mono-2-ethylhexyl phthalate and mono-isobutyl phthalate), the bromoxynil biomarker 3,5-dibromo-4-hydroxybenzoic acid, benzothiazole, and PFHxS. Additional exposures of interest included piperine, lidocaine, personal care components (3,4-dihydroxybenzoic acid and ethylparaben), the benzothiazole metabolite 2-hydroxybenzothiazole, other industrial additives such as lauryl diethanolamide, perfluorinated compounds (PFOA and PFOS), and bromoxynil. Other compounds, such as DEET, benzophenone, *N*,*N*-diphenylguanidine, caprolactam, dimethyl sebacate and tris­(2-butoxyethyl)­phosphate showed more limited occurrence. Caffeine (29–3500 ng/mL), sorbitol (47–330 ng/mL), 3,4-dihydroxybenzoic acid (nd-340 ng/mL) not only occurred in most participants, but also showed the highest absolute concentrations. Although ampicillin (nd-3500 ng/mL), erucamide (nd-24 ng/mL), fenozan (nd-13 ng/mL) and cotinine (nd-2.1 ng/mL) were rarely detected, their levels were non-negligible. Prenatal exposure to lidocaine (nd-55 ng/mL) occurs very often, and the median detected concentration (14 ng/mL) was 1 order of magnitude higher than in maternal serum (1.10 ng/mL).

Maternal urine analysis revealed consistent excretion of caffeine and 2-naphthylamine in all participants. Additional detected compounds included caffeine-related metabolites such as theophylline and theobromine, sorbitol, saccharin, acesulfame, and indole-3-acetic acid glucuronide. Interestingly, smoke exposure biomarkers were more frequently detected in urine than those of peripartum medications, such as lidocaine, hydroxylidocaine, and ampicillin. Industrial chemicals and their metabolites, including 2-hydroxybenzothiazole, Irgarol 1051, lauryl diethanolamide, *N*,*N*-dimethylaniline and cyclohexylamine, were detected in a subset of participants, alongside personal care components such as ethylparaben, 2-cyano-3,3-diphenylacrylic acid, avobenzone, DEET, and its metabolite DEET-carboxylic acid. Plastic-related metabolites including mono-2-ethylhexyl phthalate glucuronide, mono­(5-carboxy-2-ethylpentyl) phthalate, BPA-glucuronide, mono-isobutyl phthalate, and mono-2-ethylhexyl phthalate were also present.

As expected, medications administered during labor (e.g., lidocaine (nd-1200 μg/g creatinine), hydroxylidocaine (nd-720 μg/g creatinine) and ampicillin (nd-14,000 μg/g creatinine)) showed the highest urinary concentrations. Furthermore, food-related compounds, including theophylline (nd-3700 μg/g creatinine), indole-3-acetic acid glucuronide (nd-1700 μg/g creatinine) and caffeine (280–530 μg/g creatinine), as well as 2-naphthylamine (130–555,000 μg/g creatinine) and 2-hydroxybenzothiazole (nd-150 μg/g creatinine) occurred at significant levels. Less frequently detected compounds, but being excreted through urine at remarkable ranges of concentrations, entailed *N*,*N*-dimethylaniline (nd-280 μg/g creatinine), indole-3-acetic acid (nd-300 μg/g creatinine) and acesulfame (nd-220 μg/g creatinine).

### 3.2 Comparative Occurrence Across Biological Matrices

Detection frequencies varied across matrices (Figure 3), with 43 compounds identified in maternal serum, 48 in placental tissue, 45 in umbilical cord serum and 38 in urine. No compound was uniquely detected in umbilical cord serum and only a few were exclusively shared between placenta and umbilical cord serum (6) or between maternal serum and umbilical cord serum (5), indicating limited matrix-specific overlap. Additionally, placenta and urine were found to provide complementary information, as up to 19% and 45% of compounds, respectively, were uniquely present in these biospecimens. Overall, for the industrial chemicals and personal care product constituents covered in this study, placenta may provide a more representative proxy of prenatal chemical exposures than maternal serum, as it retained the highest number of unique compounds not detected in blood-based matrices. Importantly, this is not explained by analytical sensitivity differences: 52% of compounds have higher LODs in placenta than serum yet are still detected there, while 6 compounds with lower LODs in placenta are nonetheless absent, supporting a biological rather than purely analytical basis for these differences. A total of 15 compounds were consistently detected across maternal serum, placental tissue, umbilical cord serum, and urine; their concentrations profiles are shown in Figure 4. Food components reached their highest concentrations in maternal urine, consistent with renal clearance as their dominant elimination route; sorbitol, for instance, showed a median of 3200 μg/g creatinine in urine versus 26 ng/mL in maternal serum, with placental detection limited to a minority of samples and at lower levels, suggesting passage through rather than accumulation in placental tissue. Caffeine, theobromine, and theophylline were detected in nearly all participants across all four matrices at similar levels in maternal and umbilical cord serum but markedly lower in placenta, consistent with transplacental transfer rather than placental accumulation. Among pharmaceuticals, lidocaine reached its highest median concentrations in placenta (530 ng/g) and urine (600 μg/g creatinine), while ampicillin—too infrequently detected in serum and placenta for violin-based comparison—showed its highest levels in urine. Personal care product constituents and phthalate metabolites consistently peaked in placental tissue rather than in serum, most markedly for DEET (75 ng/g in placenta versus <0.2 ng/mL in maternal and umbilical cord serum). Industrial chemicals showed the strongest placental enrichment overall: benzothiazole reached a median of 1300 ng/g in placenta, roughly 60-fold higher than in either serum compartment, with 2-hydroxybenzothiazole and lauryl diethanolamide following a similar pattern. These results show that even compounds detected ubiquitously across all four compartments differ markedly in where they accumulate, reinforcing the placenta’s dual role as conduit and selective reservoir.

**Figure 3:**
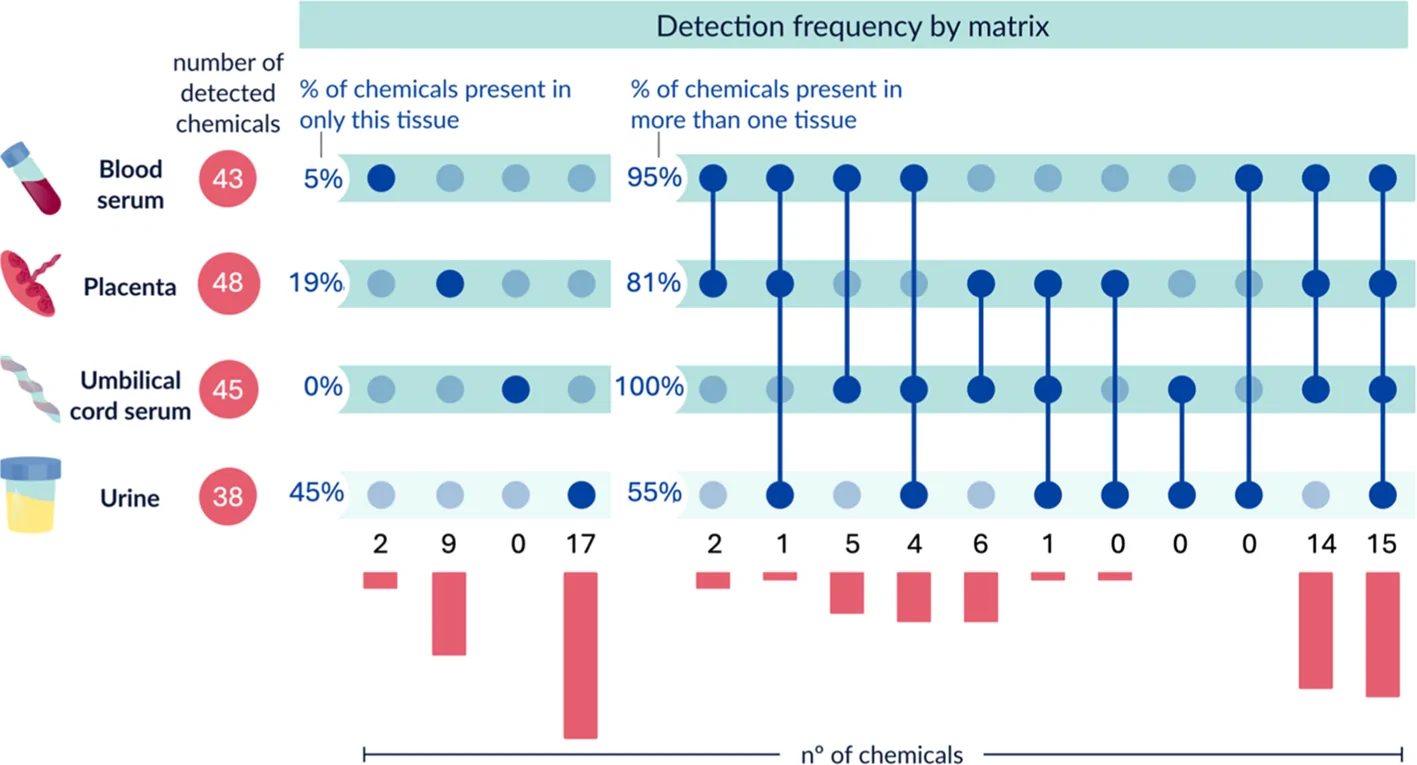
**Detection and overlap of chemicals across biological matrices**. From left to right, the figure displays the number of chemicals detected in serum, placenta, umbilical cord serum and urine, the proportion found exclusively in a single matrix, and the proportion shared across multiple matrices. The bottom panel shows the number of chemicals co-occurring across matrices, where connected dots indicate compounds detected in more than one biospecimen.

**Figure 4:**
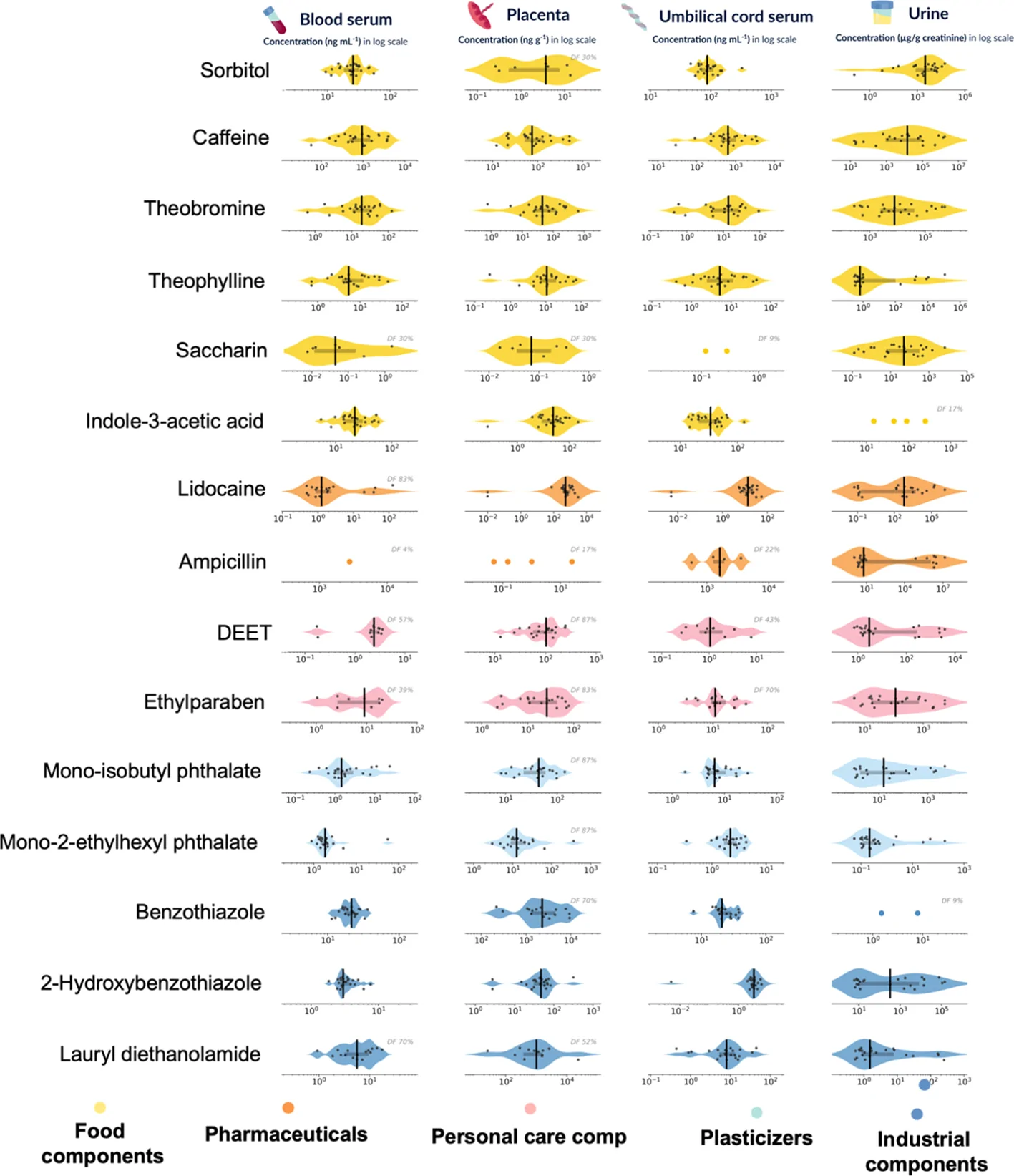
**Concentration distributions of the compounds detected across all four biological matrices**. Violin plots represent the distribution of compound concentrations detected in maternal serum, placental tissue, umbilical cord serum and maternal urine for the 15 compounds detected in every matrix. Vertical bars within each plot indicate the first quartile, the median, and the third quartile. Individual jittered points are overlaid on each violin. Compounds with a detection frequency below 20% in a given matrix are displayed as individual dots instead of violins, with the corresponding detection frequency (DF) shown as a numeric value (percentage), to account for limited detection and avoid misleading distribution shapes.

The overall pattern of compound distribution across the maternal–fetal interface is summarised in Figure 5, which depicts the detection of each compound across maternal serum, placenta, and umbilical cord serum. Of the 45 compounds detected at ≥20% frequency in at least one of these three matrices, 18 were present in all three compartments, consistent with progressive transplacental transfer. Thirteen compounds were detected exclusively in placental tissue, suggesting compartmental accumulation or retention without appreciable fetal transfer. Five compounds—including PFOS, PFHxS, and three pesticide-related structures—were detected in maternal serum and umbilical cord serum but almost absent from placental tissue. These distributional differences were further supported by inter- and intra-matrix Spearman correlation analyses. Stronger correlations were observed within matrices (e.g., between caffeine, phthalates and perfluorinated substances exposure biomarkers) (Figure S1), while inter-matrix correlations highlighted partial but incomplete concordance between maternal serum and umbilical cord serum, and placenta and umbilical cord serum, with maternal urine behaving largely independently (Figure S2).

**Figure 5:**
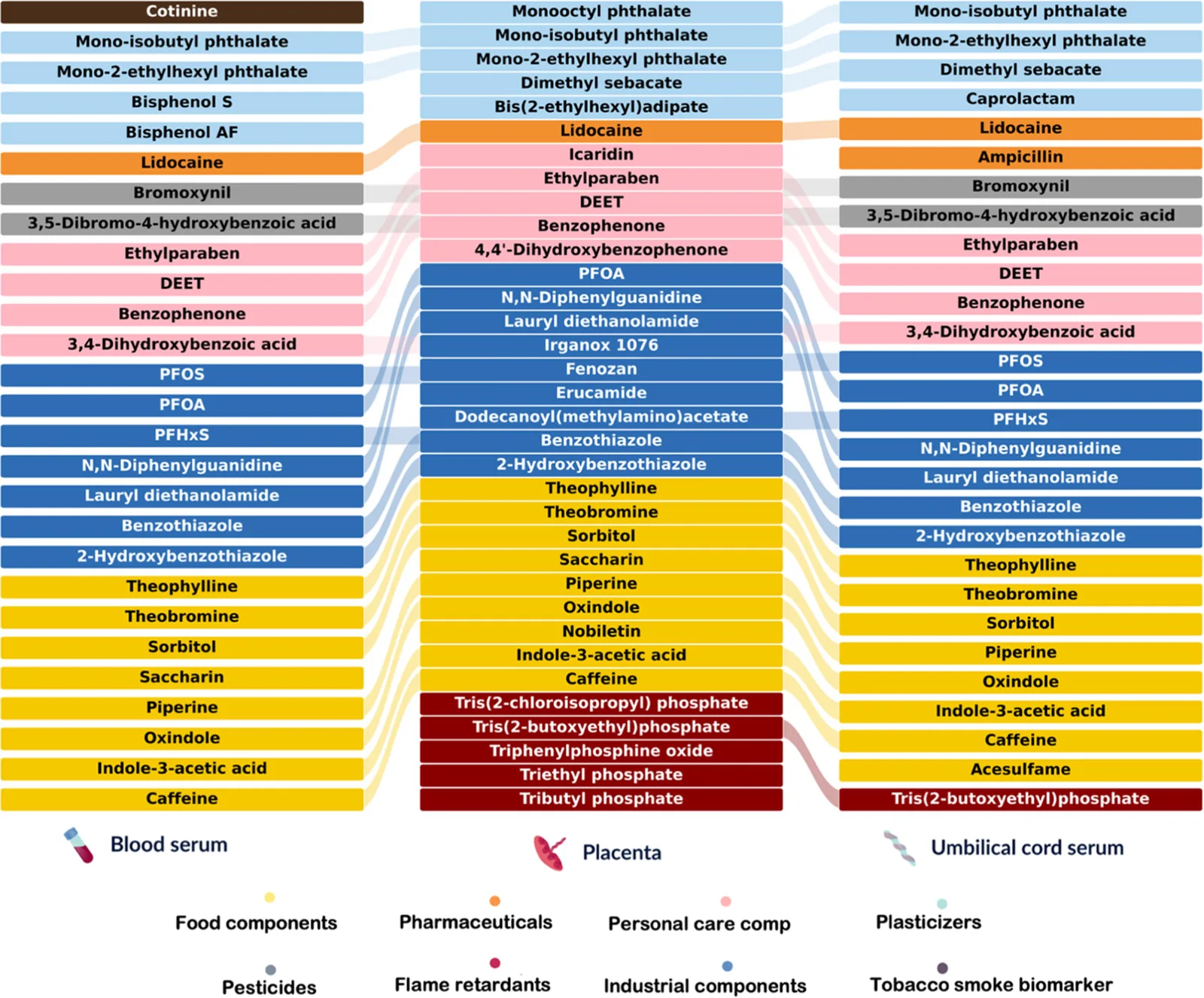
**Chemical flow across the maternal–fetal interface**. Alluvial diagram showing the detection of 45 compounds (detection frequency ≥20% in at least one matrix) across maternal serum, placenta, and umbilical cord. Each bar represents a detected compound in a given matrix; ribbons connect the same compound across consecutive matrices. Compounds are coloured by chemical family. Ribbons connecting maternal serum directly to umbilical cord serum with reduced opacity indicate compounds not detected in placental tissue, consistent with transplacental transfer without measurable placental accumulation.

Matrix-specific patterns of partitioning for individual compounds within each chemical family are further illustrated in Figures S3–S9. Most food-related compounds were detected in more than one biospecimen, except indole-3-acetic acid glucuronide, found only in urine. Caffeine and sorbitol reached similar levels in maternal and umbilical cord serum but were markedly lower in placental tissue, suggesting preferential placental passage rather than accumulation. Theobromine and theophylline showed a different pattern, with higher concentrations in placental tissue relative to both serum matrices, suggesting placental retention. Indole-3-acetic acid and oxindole were detected at broadly comparable levels across all three matrices (Figure S3). Lidocaine was present in all four biospecimens, with similar average levels in urine and placenta, and 1 order of magnitude higher in umbilical cord than in maternal serum. Hydroxylidocaine was detected at similar mean levels to lidocaine in urine, and the mean concentration of ampicillin was also markedly higher in urine than in umbilical cord serum (Figure S4). Some personal care components (e.g., 3,4-dihydroxybenzoic acid, DEET, benzophenone) exhibited comparable levels in maternal and umbilical cord serum, suggesting potential transplacental transfer. Others (e.g., ethylparaben) showed similar levels in placenta and umbilical cord serum, while a few compounds (e.g., icaridin, 4,4′-dihydroxybenzophenone) were exclusive to placenta. Several rapidly excreted compounds (e.g., avobenzone, DEET-carboxylic acid, 2-cyano-3,3-diphenylacrylic acid) were found only in urine (Figure S5). For plasticizers, five out of 11 were detected in both urine and placenta, and four in maternal and umbilical cord serum (Figure S6). When present in maternal serum, placenta, and cord serum, plasticizer levels were highest in placenta, while maternal and umbilical cord serum showed similar concentrations. Among flame retardants, tris­(2-butoxyethyl) phosphate was the only compound detected in both placenta and umbilical cord serum, with higher levels in cord serum (Figure S7). Among industrial chemicals, Irganox 1076 was the only compound detected exclusively in placental tissue, with no detectable concentrations in maternal serum, umbilical cord serum, or urine. In contrast, erucamide, fenozan, and dodecanoyl­(methylamino)­acetate showed preferential accumulation in placenta but were also detected in at least one additional matrix: erucamide and fenozan were detected at low frequency in both maternal serum and umbilical cord serum, while dodecanoyl­(methylamino)­acetate was additionally detected in maternal serum (Figure S8). Others, such as benzothiazole, 2-hydroxybenzothiazole, lauryl diethanolamide, PFOA, and *N*,*N*-diphenylguanidine, were found in placenta, but also in other matrices. Benzothiazole, 2-hydroxybenzothiazole, PFOA, and PFHxS had similar levels in maternal and umbilical cord serum while PFOS and *N*,*N*-diphenylguanidine showed lower levels in umbilical cord serum indicating partial transplacental transfer. Cotinine, a nicotine metabolite from tobacco smoke and e-cigarettes, was found in urine at levels approximately 4 orders of magnitude higher than in maternal serum (Figure S9), consistent with rapid renal clearance. Other smoke-related compounds (2-naphthylamine, 2-naphthol, and its glucuronide) were only detected in urine, likely due to their short half-lives and efficient urinary excretion.

### 3.3 Placental Retention and Transplacental Transfer

The integrated analysis of chemical presence in maternal serum, placental tissue, and umbilical cord serum allowed investigation of the molecular descriptors associated with two key toxicokinetic outcomes—placental retention and transplacental transfer—and the identification of predominant distribution patterns across the three compartments (Figure 6). Most compounds were preferentially retained in the placenta, suggesting a barrier function that limits fetal exposure. These included markers of exposure to flame retardants (triethyl phosphate, triphenylphosphine oxide, tris­(2-chloroisopropyl) phosphate), personal care product constituents (ethylparaben, icaridin, DEET, 4,4′-dihydroxybenzophenone, benzophenone), plasticizers (mono-isobutyl phthalate, mono-2-ethylhexyl phthalate, monooctyl phthalate, dimethyl sebacate, bis-2-ethylhexyl adipate), and industrial chemicals (benzothiazole, 2-hydroxybenzothiazole, *N*,*N*′-diphenylguanidine, lauryl diethanolamide, dodecanoyl­(methylamino)­acetate, fenozan, erucamide, Irganox 1076, and PFOA), along with one pharmaceutical (lidocaine) and one food-related compound (nobiletin). In contrast, a subset of chemicals with low placental retention, including theophylline, saccharin, sorbitol, indole 3 acetic acid, piperine, 3,4-dihydroxybenzoic acid and PFHxS, remained predominantly in maternal serum. A smaller number of compounds showed efficient transplacental passage, with minimal retention in placental tissue. These included the food components sorbitol, caffeine, and acesulfame, the pharmaceutical ampicillin, the pesticide bromoxynil and its metabolite 3,5-dibromo-4-hydroxybenzoic acid, the personal care product 3,4-dihydroxybenzoic acid, and the perfluorinated compound PFOS.

**Figure 6:**
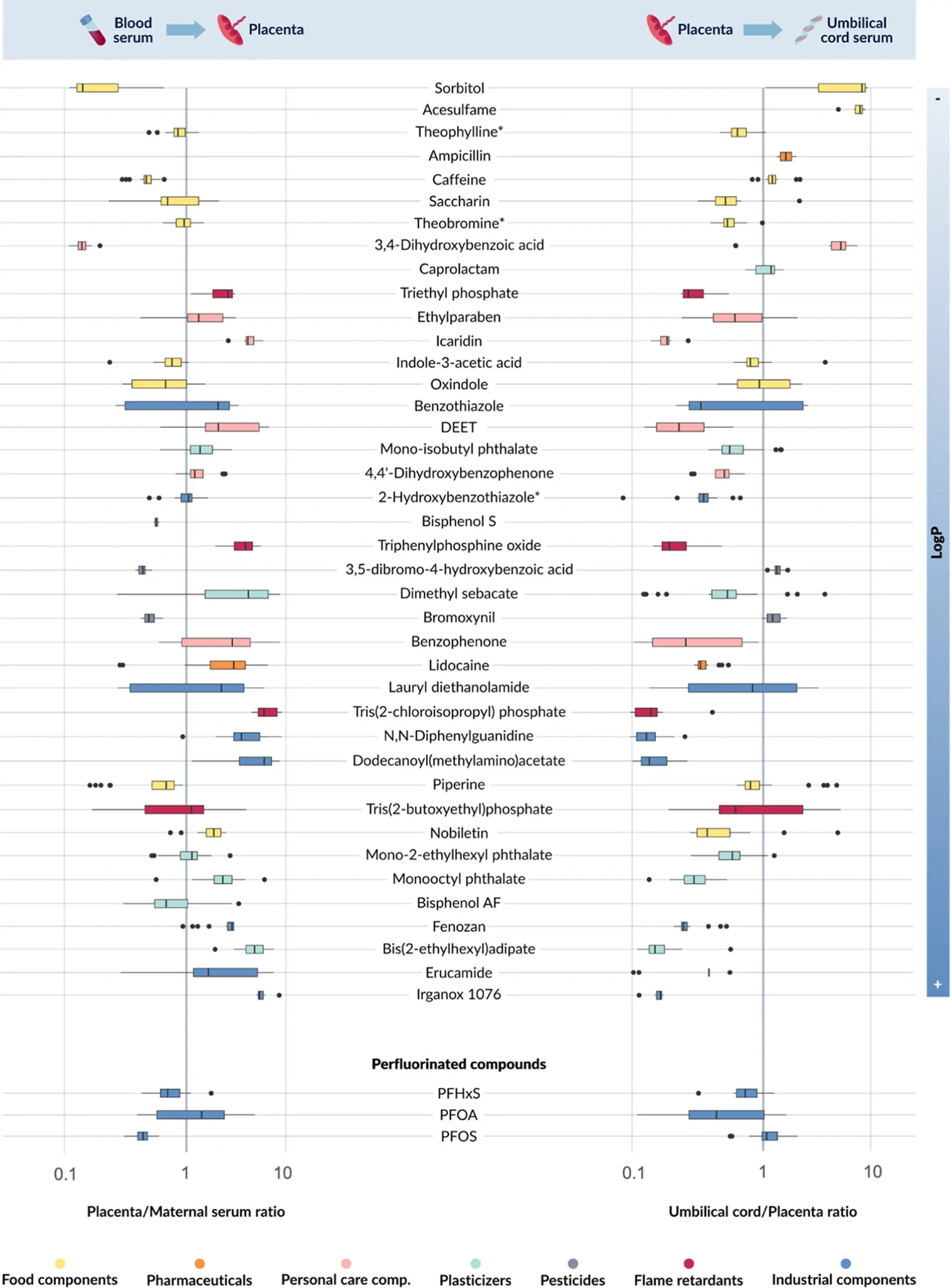
**Placental retention and transplacental transfer ratios of detected chemicals**. The left panel shows the ratio of chemical concentrations in placental tissue relative to maternal serum, indicating compound-specific placental retention. The right panel presents the ratio of umbilical cord serum to placental concentrations, reflecting the extent of transplacental transfer. Chemicals are ordered by their calculated log P values to illustrate the role of lipophilicity in distribution patterns. Perfluorinated substances (PFHxS, PFOA and PFOS) are grouped at the bottom due to their unique physicochemical behavior.

Placental retention was positively and significantly correlated with log*P* (ρ = 0.56, *p* < 0.05), while molar refractivity (ρ = 0.15), van der Waals volume (ρ = 0.15), and molecular weight (ρ = 0.13) showed weak and non-significant associations (Figure S10). Transplacental transfer showed the opposite direction for log*P* (ρ = −0.42), with weak associations observed for molar refractivity (ρ = −0.02), van der Waals volume (ρ = 0.04), and molecular weight (ρ = −0.02). Although only the correlation between log*P* and placental retention reached statistical significance—likely reflecting the limited number of compounds with detection frequency ≥70% in at least one paired matrix (*n* = 16 for placental retention, *n* = 17 for transplacental transfer)—the observed direction of log*P* and transplacental transfer is consistent with established physicochemical principles of placental transport. The relationship between each molecular descriptor and both ratios is visualised in Figure S10. The most hydrophilic compounds in the dataset, sorbitol (log*P* = −2.99) and caffeine (log*P* = −0.07), showed the highest transplacental transfer ratios, while the most lipophilic compounds generally showed lower transfer ratios and higher placental retention. However, the relationship was not absolute—piperine (log*P* = 3.48), lauryl diethanolamide (log*P* = 3.68), oxindole (log*P* = 1.22), and indole-3-acetic acid (log*P* = 1.08) all showed transfer ratios above 1 despite moderate to high log*P* values, underscoring the complexity of placental transport mechanisms and the likely involvement of active transport pathways beyond passive diffusion. PFOS, bromoxynil, and its metabolite 3,5-dibromo-4-hydroxybenzoic acid, with log*P* values ranging from 2.58 to 5.92, exhibited clear transplacental passage. Overall, our results highlight the placenta’s dual function as both a reservoir and a protective barrier. In general, lipophilic chemicals tend to accumulate in placental tissue, while more hydrophilic compounds showed higher transplacental transfer ratios. However, our data also revealed notable exceptions in both directions, highlighting that lipophilicity alone is not a sufficient predictor of placental distribution and that compound-specific transport mechanisms play an important role.

## 4 Discussion

This study provides the most comprehensive characterization to date of xenobiotics and their biotransformation products across a suite of integrated maternal–fetal biological samples, encompassing 659 compounds from diverse exposure sources. By detecting 76 unique substances across four biological matrices—maternal serum, placenta, umbilical cord serum, and urine—we reveal the extent of the prenatal chemical exposome and how compounds selectively distribute across compartments. These findings uncover compound-specific distribution patterns that reflect both passive physicochemical partitioning and, in select cases, biology that departs from passive predictions. Lipophilic molecules accumulate preferentially in placental tissue, consistent with passive partitioning into a lipid-rich compartment, while hydrophilic compounds such as caffeine, sorbitol, and the administered drug ampicillin more readily reach the fetal compartment. Notably, PFOS, PFHxS, bromoxynil and its metabolite, 3,5-dibromo-4-hydroxybenzoic acid, and the personal care component 3,4-dihydroxybenzoic acid demonstrated clear transplacental passage despite physicochemical properties that would not predict efficient passive transplacental transfer. These compounds were detected in maternal serum and umbilical cord serum in the absence of measurable placental accumulation, consistent with active carrier-mediated fetal transfer and underscoring the limits of passive diffusion as the sole explanatory mechanism for placental transport. Beyond passive transcellular diffusion, which favors small, lipophilic, and uncharged molecules, and paracellular diffusion, which allows passage of small hydrophilic molecules, the syncytiotrophoblast membrane also expresses active transporters that act in opposing directions. Efflux transporters, including *P*-glycoprotein (*P*-gp/MDR1/ABCB1) and breast cancer resistance protein (BCRP/ABCG2), limit fetal exposure by returning xenobiotics to the maternal circulation,^21^ whereas solute-carrier and organic-anion transporters, such as organic anion transporter 4 (OAT4)—implicated in the placental handling of PFOS and PFOA^22^—can move charged or amphiphilic substrates that passive diffusion alone would not predict. The latter, rather than efflux, provide the more plausible explanation for the efficient transplacental transfer of PFOS, PFHxS and bromoxynil despite their physicochemical properties. Additionally, differential plasma protein binding, particularly to albumin and fatty acid-binding proteins, constrains the free fraction of compounds available for transplacental passage,^23^ while placental biotransformation capacity (including CYP1A1, CYP2E1, and UGT enzymes) can alter the apparent distribution of parent compounds across compartments.^24^ We acknowledge that with *n* = 23 participants, definitive mechanistic attribution is not possible, and these interpretations remain hypothesis-generating. Future studies combining placental perfusion models with transporter inhibition assays would be required to confirm the specific pathways involved. Umbilical cord serum and placental tissue serve complementary roles in this context. Cord serum is the most direct indicator of chemicals actively circulating in the fetal compartment at delivery and is essential for calculating transplacental transfer ratios. However, for non-persistent compounds with short half-lives—such as parabens, phthalate metabolites, and personal care product constituents—cord serum concentrations at delivery reflect only the most recent maternal exposure episode. Placental tissue, by contrast, accumulates lipophilic and moderately persistent compounds progressively across gestation, potentially providing a more temporally integrated record of gestational exposure for this subset of chemicals. By analyzing multiple compartments simultaneously, our approach moves beyond descriptive biomonitoring to provide insight into toxicokinetic processes, which is essential for interpreting exposure in the context of developmental vulnerability and future health outcomes.

The physicochemical determinants of placental distribution observed here are broadly consistent with established principles of membrane partitioning: placental retention was significantly correlated with logP, with the most lipophilic compounds showing the greatest affinity for the lipid-rich syncytiotrophoblast, while transplacental transfer showed the inverse, non-significant relationship. Among the most hydrophilic compounds in the dataset, sorbitol (log*P* = −2.99) and caffeine (log*P* = −0.07), showed minimal placental retention yet reached the fetal circulation at comparably high concentrations, consistent with efficient passive diffusion of small, polar molecules. It should be noted that placental tissue has substantially higher lipid content than serum, and in the absence of lipid normalization, elevated placental retention ratios for highly lipophilic compounds may partly reflect thermodynamic partitioning into the lipid-rich matrix rather than active biological sequestration.

Although food-derived compounds are not the primary focus of biomonitoring studies, some—such as caffeine and theobromine—have been previously reported in maternal blood^25^ and umbilical cord serum,^26^ while sorbitol has been found in umbilical cord tissue.^27^ Our findings not only expand the biomonitoring data for several well-known food-related compounds, but also provide the first evidence of transplacental transfer for nobiletin, oxindole, sorbitol, and indole-3-acetic acid. Furthermore, our findings are consistent with previously reported transplacental transfer of acetaminophen^28^ and lidocaine,^29^ and we report for the first time higher median lidocaine concentrations in umbilical cord serum than maternal serum. The placental retention of telmisartan and transplacental passage of ampicillin are reported here for first time. Though the low detection frequencies in these cases warrant cautious interpretation, these findings are relevant because lidocaine use during labor has been associated with adverse fetal cardiotoxicity^30^ and neurobehavioral alterations,^31^ while prenatal ampicillin exposure has been linked to metabolic and gastrointestinal disorders in early life.^32^ Taken together, these results illustrate how integrated analyses can capture both environmental and clinical exposures, shedding light on their compartment-specific kinetics.

There is a substantial amount of HBM data on personal care components. In line with previous studies, we confirmed the presence of ethylparaben,^33^ benzophenones,^34^ DEET metabolites,^35^ and 2-cyano-3,3-diphenylacrylic acid^36^ in maternal serum, placenta, umbilical cord blood and urine. We also detected compounds not previously reported in maternal–fetal compartments, including icaridin and avobenzone in placental tissue, and 3,4-dihydroxybenzoic acid in cord serum. This highlights the value of wide-scope HRMS approaches to detect emerging or underregulated compounds with potential for fetal exposure. Crucially, by assessing their distribution across multiple compartments, our study extends beyond conventional biomonitoring to reveal how these chemicals partition within the maternal–fetal unit—a first step toward understanding their kinetics and potential implications for health. Maternal exposure to ethylparaben has been associated with metabolic effects in both mothers and offspring,^37,38^ while benzophenone-3 has been linked to maternal endocrine^39^ and cardiometabolic alterations,^40^ as well as impaired fetal growth^41^ and neurodevelopmental outcomes.^42^ Though evidence for 3,4-dihydroxybenzoic acid and avobenzone is still limited, both have been implicated in mechanisms involving oxidative stress^43^ and apoptosis,^44^ respectively.

Among the broad range of plasticizers humans are exposed to, we identified mono-isobutyl phthalate in placenta and, for first time, detected dimethyl sebacate, bis­(2-ethylhexyl) adipate, and caprolactam across maternal serum, placenta and cord serum—a novel observation not previously reported in humans. These findings expand previous reports on BPA and phthalates,^45,46^ suggesting that both legacy and replacement plasticizers may more readily cross the placental barrier than previously recognized. Mechanistic studies have showed that BPA and its analogs can disrupt placental function through oxidative stress, apoptosis, and gut–placenta axis alterations,^47,48^ as well as triggering epigenetic modifications in cord blood.^49^ While evidence on transplacental transfer of plasticizers remains largely confined to placental perfusion models^50^ or animal models,^45,51^ extensive epidemiological data link prenatal bisphenol and phthalate exposure to maternal endocrine and metabolic disruptions,^52–54^ impaired fetal growth and preterm birth,^55,56^ and adverse child developmental outcomes.^57^ The detection of these compounds in both placental and fetal compartments underscores the need to re-evaluate the presumed safety of plasticizers and to prioritize them for toxicological assessment in human-relevant pregnancy models.

Here, we report the presence of bromoxynil and its metabolite 3,5-dibromo-4-hydroxybenzoic acid in both maternal and umbilical cord serum, and acibenzolar-*S*-methyl in placenta—compounds for which transplacental transfer has not been previously characterized. These findings build on prior biomonitoring studies^58,59^ and expand the known range of bioavailable pesticide residues during pregnancy, highlighting the potential for co-exposure to pesticide-metabolite mixtures at early life stages.

Transplacental transfer efficiency of organophosphate esters has been largely investigated and linked to their physicochemical properties,^60^ yet far less is known about newer or less studied compounds. We report for the first time that triethyl phosphate, triphenylphosphine oxide, and tris­(2-chloroisopropyl) phosphate can reach placenta and cord serum, and we confirmed transplacental transfer of tributyl phosphate and tris­(2-butoxyethyl) phosphate, in line with prior evidence.^61^ Prenatal exposure to these organophosphate flame retardants has been associated with maternal metabolic alterations,^62^ impaired fetal growth,^63^ and early-life immune^64^ and metabolic^65^ disorders. The frequent detection of multiple flame retardants in placenta and cord serum highlights the need to incorporate this class of chemicals more systematically into HBM programs during pregnancy.

While the transplacental transfer of PFOS and PFOA has been previously studied, earlier estimates relied on maternal and neonatal samples,^66^ or maternal and umbilical cord serum pairs,^67^ leading to variability in placental ratio calculations. In our study, we detected PFOS, PFOA and PFHxS in both maternal and umbilical cord serum, providing direct evidence of maternal–fetal transfer. Of these, PFOA was also detected in placental tissue, allowing its placental retention and transfer efficiency to be quantified directly; PFOS and PFHxS were not measurably retained in placenta, indicating transfer to the fetal compartment without appreciable placental accumulation. Evidence has linked prenatal exposure to PFOS and PFOA with gestational metabolic^68^ and immune disturbances,^69^ as well as impaired fetal growth.^15^ Recent multi-omics studies have further emphasized the role of prenatal exposures in shaping the molecular underpinnings of childhood obesity and metabolic dysfunction.^70^

Cotinine was detected in both maternal and umbilical cord serum, along with urine, confirming tobacco smoke exposure and aligning with previous biomonitoring data linking prenatal and postnatal exposure to adverse health outcomes.^71,72^ This underscores the urgency of eliminating tobacco exposure during pregnancy. We also confirmed the presence of smoke-related biomarkers such as *N*,*N*-dimethylaniline and cyclohexylamine, consistent with previous studies.^73,74^ Among industrial chemicals, erucamide and fenozan were detected in blood,^75,76^ and we report for the first time that these, along with lauryl diethanolamide, *N*,*N*-dimethyltetradecylamine-*N*-oxide, and dodecanoyl­(methylamino) acetate, can cross the placenta. We also identified benzothiazoles in placental tissue and 2-hydroxybenzothiazole in umbilical cord serum for the first time. Together, these findings suggest that several poorly characterized industrial additives and transformation products can reach the fetal environment and may warrant prioritization for toxicological testing.

A major strength of this study lies in the simultaneous analysis of a suite of integrated maternal–fetal biological samples. This integrative design enables within-subject comparisons of chemical distribution across compartments, offering a rare and comprehensive view of xenobiotic transfer during pregnancy. By capturing both persistent and transient exposures, we were able to classify compounds according to their toxicokinetic behavior—whether they persist in maternal circulation, accumulate in the placenta, or cross into the fetal environment. Placental tissue, often underrepresented in biomonitoring studies, proved essential for detecting long-term maternal exposures not fully captured by blood or urine, as it accumulates compounds from maternal circulation across gestation. However, depending on the physicochemical properties of the compound, placental retention may simultaneously reflect barrier role—compounds sequestered in placental tissue may not reach the fetal circulation and would therefore be absent from cord serum. This multi-matrix analytical design, combining simultaneous sampling of maternal serum, placental tissue, umbilical cord serum, and urine within the same individual, represents a scalable blueprint for future exposome-wide studies during pregnancy.

### 4.1 Limitations

Although 659 chemicals were screened, our analytical approach is based on a targeted library of pre-defined compounds and therefore captures a defined subset of the full chemical exposome; unknown or library-absent compounds are not detected, and the approach should be understood as a wide-scope targeted characterization rather than a fully untargeted exposomic analysis. Consequently, our final evaluation focused on 76 compounds that were confidently detected and annotated at Level 1, potentially underestimating the full scope of maternal–fetal chemical exposure. Within the screened library, detection is inherently biased toward compounds present at higher concentrations, and trace-level contaminants may fall below the limits of detection even when present. Although a suspect screening workflow was applied to identify phase II metabolites (glucuronide conjugates) of library compounds, classified at Level 2b confidence according to the Schymanski scale, broader untargeted feature annotation—extending Level 2 identification to HRMS features not linked to a known parent compound in our library—was not systematically performed, potentially further underestimating the number of detectable compounds within the existing dataset. Future work extending such annotation would complement the current findings and may reveal additional compounds of interest. Furthermore, the analytical approach is restricted to compounds amenable to LC–MS analysis and therefore does not capture persistent organic pollutants (POPs), including polychlorinated biphenyls (PCBs), organochlorine pesticides, and polybrominated diphenyl ethers (PBDEs), which are ubiquitously detected in human placenta and cord blood and are typically quantified by GC–MS. Integration of complementary GC-MS-based platforms in future studies would substantially expand the chemical space covered and provide a more complete characterization of the maternal–fetal chemical burden.

The study is cross-sectional, capturing a single time point per matrix, which may not accurately reflect long-term exposure, especially for non-persistent compounds with short half-lives. This limitation is compounded by an inherent temporal asymmetry between matrices: umbilical cord serum reflects fetal chemical exposure at a single time point—delivery—whereas placental tissue provides a more temporally integrated, though spatially and matrix-averaged, record of gestational exposure for lipophilic and moderately persistent compounds. This temporal mismatch limits the direct comparability of concentrations and derived transfer and retention ratios across compartments and should be considered when interpreting the compartmentalization patterns reported here. This is particularly relevant for compounds at opposite ends of the persistence spectrum: for short-lived compounds such as caffeine—whose half-life is prolonged to approximately 10–15 h in late pregnancy due to reduced CYP1A2 activity—maternal serum concentrations at delivery reflect very recent exposure, whereas for highly persistent compounds such as PFOS (serum elimination half-life of approximately 4–5 years), serum and placental levels more closely approximate a steady-state distribution, making the derived ratio more directly interpretable as compartmental partitioning rather than a transient exposure snapshot.

The wide-scope targeted approach was limited by the availability of analytical standards and spectral libraries, which constrained the annotation of some detected features. Moreover, detection limits and matrix effects may vary across biological samples, potentially affecting cross-compartment comparisons. This study was conducted in a single cohort recruited at a single centre in Tarragona, Spain (*n* = 23 mother–infant dyads), which, while sufficient for an exploratory characterization of the prenatal chemical exposome, limits the generalizability of the compartmentalization patterns reported here to other populations with different dietary habits, geographical exposures, and demographic profiles. Replication in independent cohorts with larger and more diverse sample sizes will be necessary to confirm and extend these findings.

Most compounds were semi-quantified using a surrogate standard (clothianidin-*d*_3_) applied uniformly across all analytes, in the absence of compound-specific isotopically labelled standards. Although full validation data indicates that differential recovery across matrices is unlikely to substantially bias the reported distribution patterns for most compounds, semi-quantification introduces residual uncertainty in absolute concentration estimates and in derived transfer and retention ratios, particularly for non-validated compounds, which should therefore be interpreted as indicative of directional tendencies rather than precise quantitative transfer rates. The semi-quantification model was built separately for each matrix and therefore inherently incorporates matrix-specific ionization effects. Among validated compounds, inter-matrix recovery differences were modest, and the urine method’s recovery performance was previously established;^16^ however, for compounds lacking experimental recovery data, recovery was assumed to be 100%, as no compound-specific correction factor could be derived; and residual uncertainty in the derived placental retention and transplacental transfer ratios for non-validated compounds cannot be excluded.

Furthermore, lipid content was not measured in either matrix in the present study. Given the substantially higher lipid content of placental tissue relative to serum, the placental retention ratios reported here for lipophilic compounds should be interpreted with caution, as they may partly reflect thermodynamic partitioning rather than active biological retention. Additionally, no sampling blanks were collected during the field sampling procedure, meaning that contamination potentially introduced by sampling equipment—including BD Vacutainer tubes, polypropylene containers, and PBS solution used for placental tissue rinsing—cannot be formally excluded. Compounds detected at high frequency with low inter-individual variability in both maternal and umbilical cord serum, such as benzothiazole, Irganox 1076, and some phthalate metabolites, were also detected in procedural blanks in this study (Table S4) and are structurally consistent with chemical classes—rubber vulcanization accelerators, plastic antioxidants, and plasticizers—documented as contaminants of specimen-collection and laboratory plasticware,^77,78^ and should therefore be interpreted with appropriate caution. Additionally, 3,5-dibromo-4-hydroxybenzoic acid is the primary metabolite of bromoxynil, though a minor contribution from background contamination cannot be entirely excluded. Finally, urinary concentrations were normalized to creatinine to account for urine dilution; while this is the standard approach in epidemiological biomonitoring studies and facilitates cross-study comparability, glomerular filtration rates and muscle metabolism undergo dynamic changes during late pregnancy, which may introduce additional variability in creatinine-normalized concentrations. Specific gravity represents an alternative normalization approach, though its application in cohort studies has been less consistent in the literature. Despite these limitations, this work redefines how prenatal exposure is assessed, demonstrating that relying solely on maternal blood or urine underestimates fetal exposure to structurally diverse and biologically active xenobiotics. The widespread presence of underregulated and emerging compounds in fetal compartments calls for a fundamental rethinking of how we monitor, prioritize, and regulate chemical exposures during pregnancy.

## 5 Implications for Prenatal Exposure Assessment

Using wide-scope LC-HRMS, we show that environmental chemicals exhibit compound-specific partitioning behaviors across the maternal–fetal interface, governed in part by physicochemical properties. Lipophilic compounds preferentially accumulated in placental tissue, whereas more hydrophilic chemicals such as caffeine, sorbitol, and ampicillin more readily reached the fetal circulation via passive diffusion. However, several compounds—including PFOS, PFHxS, and bromoxynil—exhibited efficient transplacental passage despite physicochemical properties inconsistent with passive diffusion, implicating active carrier-mediated transport as an additional determinant of fetal exposure. These results demonstrate that fetal exposure cannot be inferred from any single biospecimen and establish a scalable, systems-level approach for prioritizing chemicals based on their likelihood of transplacental transfer. This framework extends exposome analysis beyond descriptive biomonitoring toward mechanistic understanding of chemical transport during pregnancy and is readily adaptable to exposome-wide association studies in human cohorts.

## Supplementary Material





## Data Availability

The data that support the findings of this study have been uploaded to Zenodo (0.5281/zenodo.17340310) and will be accessible upon publication. A preprint of the manuscript has been published (10.21203/rs.3.rs-8085020/v1).

## Notes

A dedicated supplementary methods document provides full procedural details for the analytical workflow, organised in four sections: **SI-1** (Chemicals and reagents): full list of solvents, reagents, standards, and consumables with suppliers, grades, and purities; **SI-2** (Sample preparation): surrogate standard spiking volumes and equilibration procedure for all three matrices, full placental tissue extraction and SPE clean-up protocol (sorbent composition, conditioning, loading, drying, and elution steps), and urine filtration protocol with rationale for method selection; **SI-3** (Data evaluation and quantification): matrix-matched calibration curve construction at nine concentration levels, handling of endogenous analytes in pooled QC samples, matrix-effect correction factors, semi-quantification model application, and surrogate standard normalization; **SI-4** (QA/QC and data analysis): contamination control measures during sample pretreatment, blank strategy rationale (Milli-Q water across all matrices), model-based LOD threshold derivation, inter-matrix recovery ratios for 52 validated compounds, software details (RStudio 2024.12.1 and Python v3.12.3), and physicochemical descriptor selection rationale (logP, molecular weight, molar refractivity, and van der Waals volume). (Table S1) full analytical validation data for the serum extraction method, including recovery, matrix effects, reproducibility, and limits of detection; (Table S2) full analytical validation data for the placental tissue extraction method, including recovery, matrix effects, reproducibility, and limits of detection; (Table S3) List of reference standards included in the calibration curves, including compound name, molecular formula, exact mass, adduct, and InChIKey; (Table S4) concentrations of compounds detected in blanks across all four matrices; (Table S5) limits of detection for all screened compounds, with indication of LOD type (“Experimental” or “Model-based”); (Table S6) estimated limits of detection for compounds screened but not detected in any sample, derived using the matrix-specific semi-quantification model; (Table S7) complete list of detected compounds with detection frequencies, concentration ranges, and identification confidence levels (Schymanski Level 1 or Level 2b) across maternal serum, placental tissue, umbilical cord serum, and maternal urine (XLSX)
(Figure S1) intra-matrix Spearman correlation heatmaps of detected compounds within each biological matrix (maternal serum, placental tissue, umbilical cord serum, and maternal urine); (Figure S2) inter-matrix Spearman correlation heatmaps between all paired biological matrices; (Figure S3–S9) matrix-specific concentration distributions by chemical family (food components, pharmaceuticals, personal care components, plasticizers, flame retardants, industrial components, and tobacco-related compounds); (Figure S10) scatter plots showing Spearman correlations between four molecular descriptors (log*P*, molecular weight, molar refractivity, and van der Waals volume) and placental retention ratio (panel A) and transplacental transfer ratio (panel B) (PDF)
