# Supplementary material for: Matrix-Resolved Profiling of the Prenatal Chemical Exposome Reveals Selective Placental Retention and Fetal Transfer
Source: Environ Sci Technol. 2026 Sep 24;60(39):27562–78. doi: 10.1021/acs.est.6c02272 (PMC13648230; doi:10.1021/acs.est.6c02272)
Supplement: Supplementary file 1 [file es6c02272_si_002.pdf]

Supporting Information for the Article:

Matrix-resolved profiling of the prenatal chemical exposome reveals selective placental retention and fetal transfer

Authors: Montse Marquès[1,2,3*], Ruben Gil-Solsona[1], Ana González-Ruiz[1,4,5], Esteban Restrepo-Montes[1,6], Ana Megía[5,7], Monica Ballesteros[5,8], José L. Domingo[4,5], Ana Navas-Acien[3], Gary W. Miller[3], Adrian Covaci[2], Pablo Gago-Ferrero[1]

[1] *Institute of Environmental Assessment and Water Research (IDAEA-CSIC), Barcelona, Spain.*
[2] *Toxicological Centre, Department of Pharmaceutical Sciences, University of Antwerp, Universiteitsplein 1, Antwerp, Belgium.*
[3] *Department of Environmental Health Sciences Columbia University Mailman School of Public Health New York, New York, NY.*
[4] *School of Medicine, Universitat Rovira i Virgili, Laboratory of Toxicology and Environmental Health, Reus, Spain.*
[5] *Institut d'Investigació Sanitària Pere Virgili (IISPV), Doctor Mallafré Guasch 4, 43005 Tarragona, Spain.*
[6] *PhD program "Analytical chemistry and Environment", Universitat de Barcelona*
[7] *Department of Endocrinology and Nutrition, Research Unit, University Hospital of Tarragona Joan XXIII-Institut d'Investigació Sanitària Pere Virgili (IISPV), Tarragona, Spain.*
[8] *Department of Obstetrics and Gynecology, University Hospital of Tarragona Joan XXIII. Institut d'Investigació Sanitària Pere Virgili (IISPV), Tarragona, Spain.*

Supplemental methods:

SI-1 Chemical and reagents

Methanol (HPLC-grade), acetonitrile (HPLC-grade), water (HPLC-grade), formic acid (>99% purity), ammonium acetate, and ammonium formate (≥99.0% purity) were purchased from Merck (Darmstadt, Germany). Ethyl acetate 99.6% was purchased from Acros Organics (Thermo Fischer Scientific). Ultrapure water was obtained by a Milli-Q purification system (Arium, PRO-VFT, Sartorius, Göttingen, Germany). Ammonia solution (32%) was purchased from VWR Chemicals (France). Citric acid anhydrous and tri-sodium citrate 2-hydrate were purchased from Scharlab (Barcelona, Spain). Standards used in the preparation of the calibration curve and recovery experiments were obtained from Sigma-Aldrich (Steinheim, Germany) and LGC Standards (Barcelona, Spain). Captiva 3 mL Non-Drip filter cartridges were bought from Agilent (Madrid, Spain). For SPE cartridge preparation, solid phase extraction (SPE) empty cartridges, frits, Sepra ZT (30 µm, 85 Å) powder, Sepra ZTL-WCX (100 µm, 300 Å) powder, and Sepra ZTL-WAX (115 µm, 330 Å) powder were purchased from Phenomenex (Madrid, Spain), and Isolute ENV+ was purchased from Biotage (Uppsala, Sweden).

SI-2 Sample Preparation

Surrogate standard spiking (clothianidin-d_3). A clothianidin-d_3 stock solution prepared in methanol was used as a surrogate standard and added to every sample prior to extraction to control for extraction performance. For serum, 30 µL of the 1000 ng/mL stock was added to 150 µL of sample, followed by 450 µL of acetonitrile (final concentration ~47.5 ng/mL). For urine, 50 µL of the 1000 ng/mL stock was added to 1 mL of sample (final concentration ~47.5 ng/mL). For placental tissue, where the surrogate cannot be homogeneously incorporated into a liquid matrix, the methanolic stock was applied directly onto the fresh tissue, which was then left open at room temperature for ~60 min to allow the methanol to evaporate and the surrogate to distribute throughout the tissue before extraction (target ~50 ng/g of fresh tissue).

Placental tissue extraction and clean-up: Placental tissue (~200 mg) was homogenized in a tissuelyzer with 1 g of zirconium oxide beads and 1 mL of citrate buffer:acetonitrile (1:1, v/v) at 50 Hz for 2 min. After centrifugation (10,000 rpm, 10 min), the supernatant was recovered and the extraction was repeated twice; the three extracts were combined. The pooled extract was diluted with 100 mL of Milli-Q water to reduce the acetonitrile content below 5% and loaded onto home-made SPE cartridges containing 0.2 g Sepra ZT, 0.1 g Sepra ZTL-WCX, 0.1 g Sepra ZTL-WAX, and 0.15 g Isolute ENV+, preconditioned with 6 mL of methanol followed by 6 mL of Milli-Q water (pH 6.5). [1] After drying with air for 15 min, analytes were eluted

with 4 mL of ethyl acetate:methanol (1:1, v/v) containing 2% ammonia, followed by 2 mL of ethyl acetate:methanol (1:1, v/v) containing 1.8% formic acid. Eluates were evaporated to dryness under a gentle nitrogen stream and reconstituted in ~200 µL of methanol:water (1:1, v/v), with the final volume adjusted proportionally to the initial tissue mass to achieve a 1× concentration factor. This factor was selected after evaluating 1×, 2×, and 5× during method optimization. Validation details al available in **Table S2**.

Urine sample preparation. Urine (1 mL) was centrifuged at 3,500 rpm for 5 min. The supernatant was passed directly through a Captiva Non-Drip filtration cartridge, and 475 µL of the filtrate was mixed with 25 µL of methanol in a chromatographic vial for analysis. This minimal, filtration-based preparation was selected, after evaluation of alternative approaches, for its adequate removal of particulate matter and high-molecular-weight matrix components while preserving acceptable ionization and instrumental sensitivity [2]. Urinary creatinine was determined in parallel following a previously published protocol [3] to correct for urine dilution.

SI-3 Data evaluation and quantification

For each matrix, matrix-matched calibration curves were prepared using pooled QC samples (equal volumes of all deproteinized serum samples, all extracted urine samples, and aliquots of a single placental tissue sample extracted as described above). Different volumes of a standard mixture of 80 reference standards (**Table S3**) was added to 250 µL inserts, evaporated to dryness under a gentle nitrogen stream, and reconstituted with 200 µL of either the matrix QC pool or methanol:water (5:95, v/v) for the parallel solvent curve, yielding calibration points at 0, 0.1, 0.5, 1, 2.5, 5, 10, 25, 50, and 100 ng/mL. When the analyte was present in the matrix QC pool, the 0 ng/mL response was subtracted from all calibration points, linearity was verified ($R^2 \geq 0.98$), and the corrected slope was used provided at least four points remained within the linear range. For compounds present at 10–50 ng/mL in the matrix QC pool but absent from the solvent curve, the solvent calibration slope was applied with a matrix-effect correction factor derived from the matrix-to-solvent response ratio at 100 ng/mL. For compounds whose endogenous levels precluded reliable matrix-effect determination, a matrix-specific semiquantification model based on the matrix-matched curves was applied, incorporating matrix-specific ionization effects for each biological matrix. Peak areas were normalized to the clothianidin-d_3 surrogate to correct for between-sample variability in extraction recovery and instrument response.

SI-4 QA/QC and data analysis

Contamination control. To minimize background contamination during sample pretreatment, all glassware was baked in a muffle furnace at 450 °C to combust residual organics, and remaining labware was restricted to polypropylene or glass and pre-rinsed with sample solvent before use. Solvents and reagents were of LC–MS grade or higher purity, work surfaces were cleaned with methanol/isopropanol before each session, nitrile gloves were worn throughout, and sample preparation was performed in a dedicated analytical chemistry laboratory separated from potential contamination sources.

Blank strategy. Milli-Q water was used as the blank across all four matrices because certified blank human matrices free of the target analytes are not commercially available for placental tissue or umbilical cord serum, and zero-background status cannot be guaranteed for ubiquitous contaminants in commercial serum or urine blanks. Blanks (n = 5) were processed in the same cryovials used for biological sample storage to capture container-derived contamination.

Model-based LOD threshold. From the performance of validated standards, the practical detection threshold corresponded to a signal area of ~90,000 counts (positive mode) and ~75,000 counts (negative mode), below which peaks were not reliably detected. Compounds for which the semiquantification model could not generate reliable LOD estimates owing to insufficient structural coverage are indicated as "Outside model coverage" in **Table S6**.

Inter-matrix recovery. For the 52 compounds validated in all three matrices, median inter-matrix recovery ratios were 1.17 (serum/placenta), 0.98 (urine/placenta), and 0.82 (urine/serum).

Software. Data processing and visualisation used R v4.3.1 (*dplyr, tidyr, tibble, readxl* for processing; *Hmisc* for Spearman correlations; *webchem* for PubChem descriptor retrieval; *ggplot2, ggrepel, cowplot, patchwork* for visualisation) and Python v3.12.3 (*matplotlib, numpy, pandas*).

Selection of physicochemical descriptors. Four descriptors were selected for their established mechanistic relevance to membrane partitioning and transplacental transport: logP, molecular weight, molar refractivity, and van der Waals volume. LogP and molecular weight are primary determinants of passive diffusion across lipid bilayers; van der Waals volume captures the molecular bulkiness that sterically constrains membrane permeation; and molar refractivity reflects the combined contribution of molecular size and polarizability.

REFERENCES:

[1] Gil-Solsona, R., Nika, M.-C., Bustamante, M., Villanueva, C. M., Foraster, M., Cosin-Tomás, M., Alygizakis, N., Gómez-Roig, M. D., Llurba-Olive, E., Sunyer, J., Thomaidis, N.

S., Dadvand, P. y Gago-Ferrero, P. The Potential of Sewage Sludge to Predict and Evaluate the Human Chemical Exposome. *Environ. Sci. Technol. Lett.* **8**, 1077–1084 (2021).

[2] Gutiérrez-Martín, D., Restrepo-Montes, E., Golovko, O., López-Serna, R., Aalizadeh, R., Thomaidis, N. S., Marquès, M., Gago-Ferrero, P. y Gil-Solsona, R. Comprehensive profiling and semi-quantification of exogenous chemicals in human urine using HRMS-based strategies. *Anal. Bioanal. Chem.* **415**, 7297–7313 (2023).

[3] González, N., Pàmies, C., Martinez, P., Martí, L., Domingo, J. L., Nadal, M. y Marquès, M. Effects of an organic diet intervention on the levels of organophosphorus metabolites in an adult cohort. *Food Research International* **173**, 113354 (2023).

Supplemental figures:

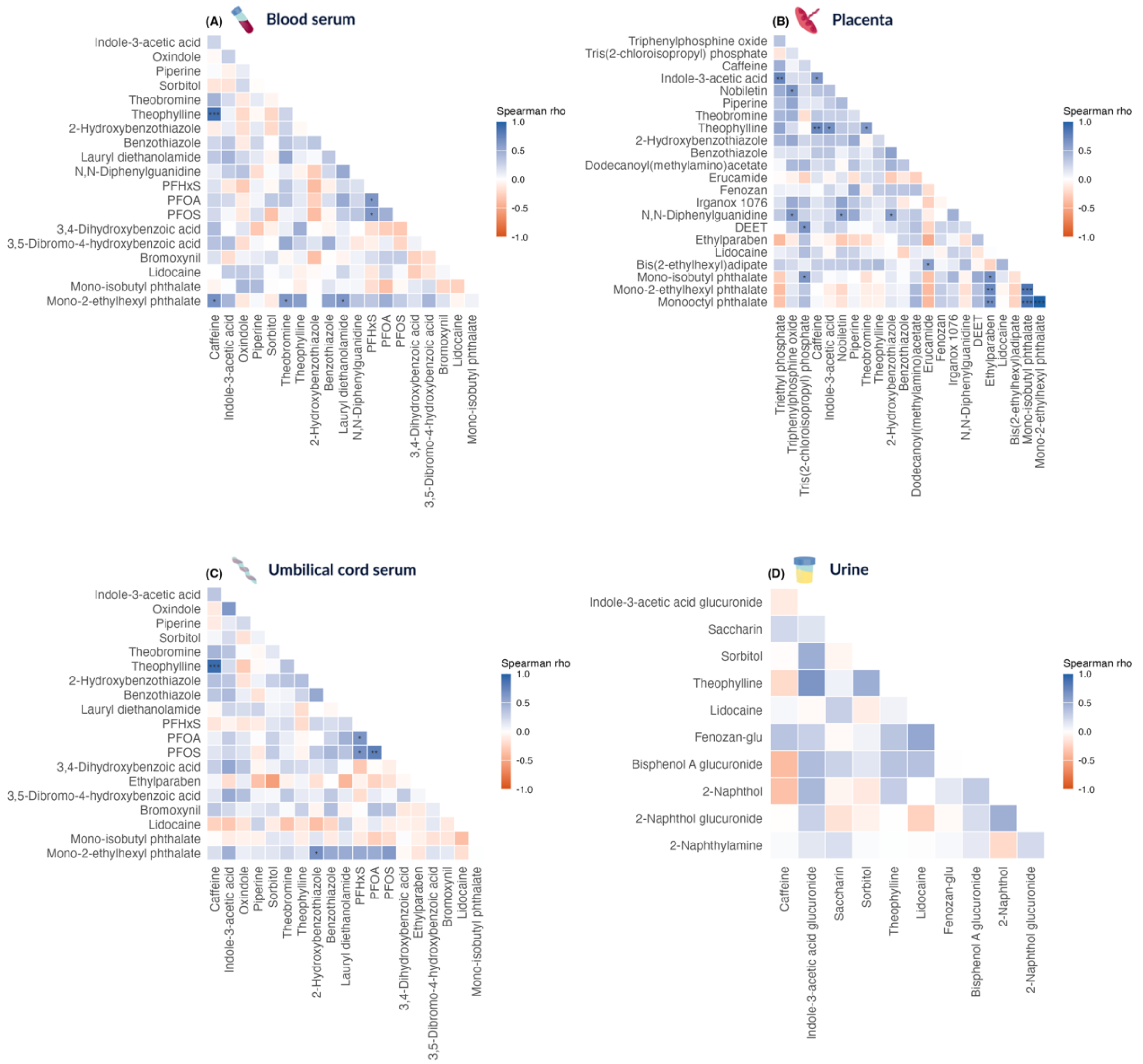

Figure S1. Intra-matrix Spearman correlation heatmaps of detected compounds across biological matrices. Spearman correlation coefficients (ρ) are shown for pairs of compounds detected within (A) blood serum, (B) placental tissue, (C) umbilical cord serum, and (D) urine. Only compounds with a detection frequency ≥70% within each matrix were included. Positive correlations are shown in blue and negative correlations in red. Significance levels are indicated as follows: * $p < 0.05$, ** $p < 0.01$, *** $p < 0.001$, all corrected for multiple testing using the BH-FDR procedure. Blank cells indicate pairs for which no statistically significant correlation was retained after BH-FDR correction.

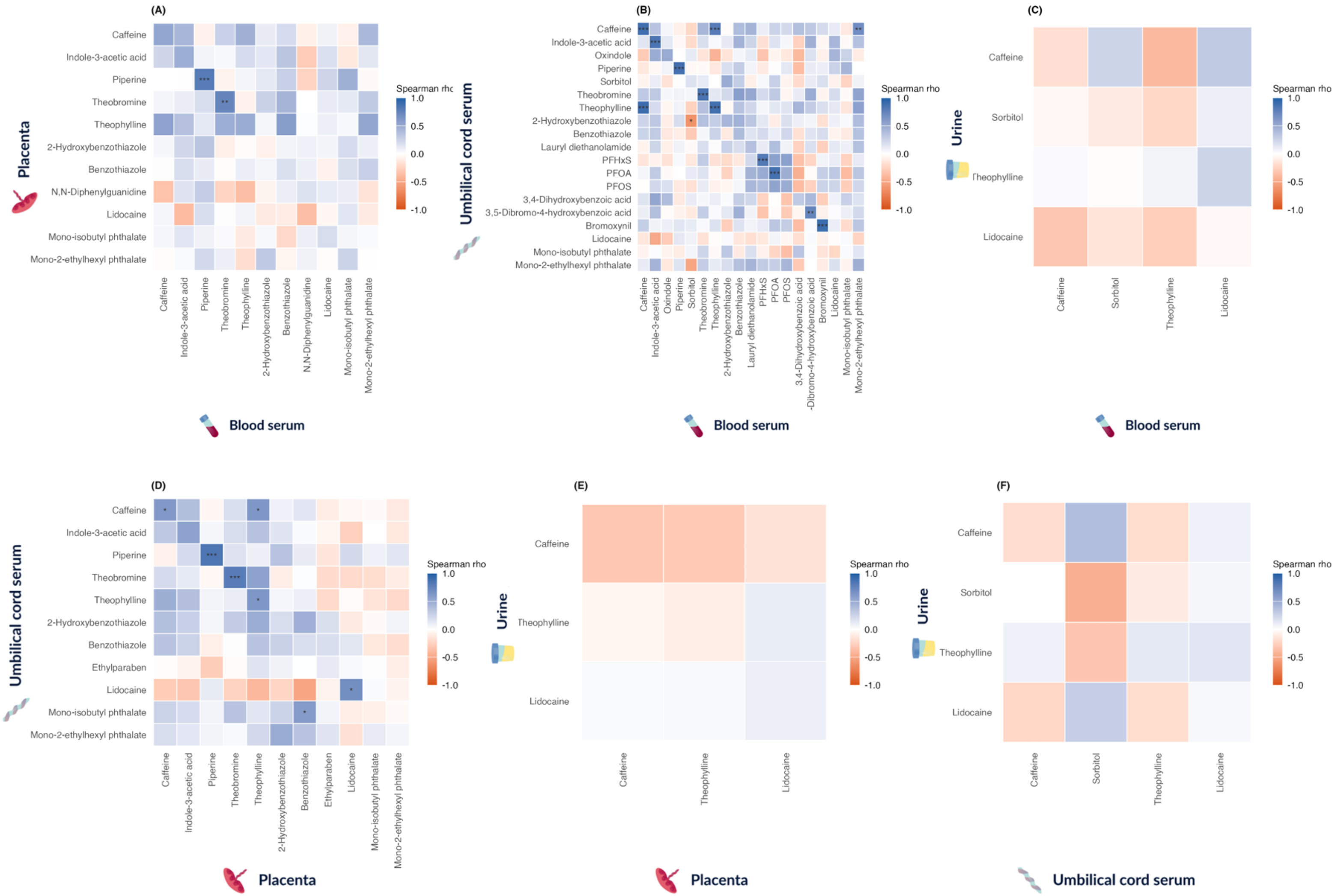

Figure S2. Inter-matrix Spearman correlation heatmaps between paired biological matrices.
Spearman correlation coefficients (ρ) are shown for all pairs of compounds detected in both matrices of each pair, with detection frequency ≥70% in each matrix. Panels show correlations between: (A) placenta and blood serum (n=11), (B) umbilical cord serum and blood serum (n=19), (C) urine and blood serum (n=4), (D) umbilical cord serum and placenta (n=11), (E) urine and placenta (n=3), and (F) urine and umbilical cord serum (n=41). Positive correlations are shown in blue and negative correlations in red. Significance levels are indicated as follows: * $p < 0.05$, ** $p < 0.01$, *** $p < 0.001$, all corrected for multiple testing using the BH-FDR procedure. Matrix pairs with $n \leq 4$ compounds should be interpreted with caution.

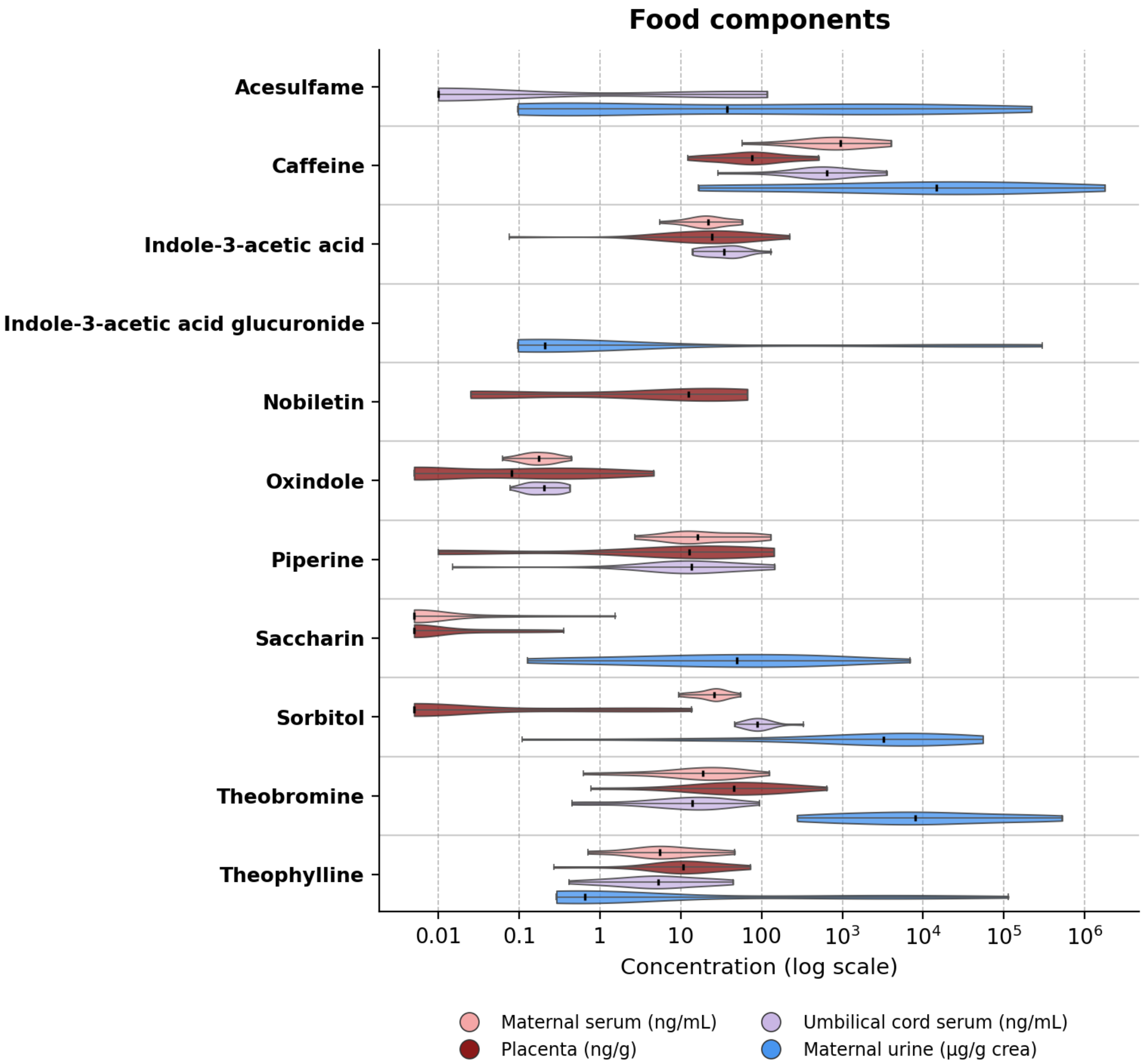

Fig. S3. Violin plots of food components concentrations across biological matrices.
Each distribution represents the $\log_{10}$-transformed concentrations (*ng mL^{-1} for serum, ng g^{-1} for placenta and µg g^{-1} creatinine for urine) of food components in different biological matrices. Violin widths indicate the density of observations, and internal lines represent the 25th, 50th (median), and 75th percentiles. Only chemicals with detection frequencies ≥ 20% in the matrices are shown.

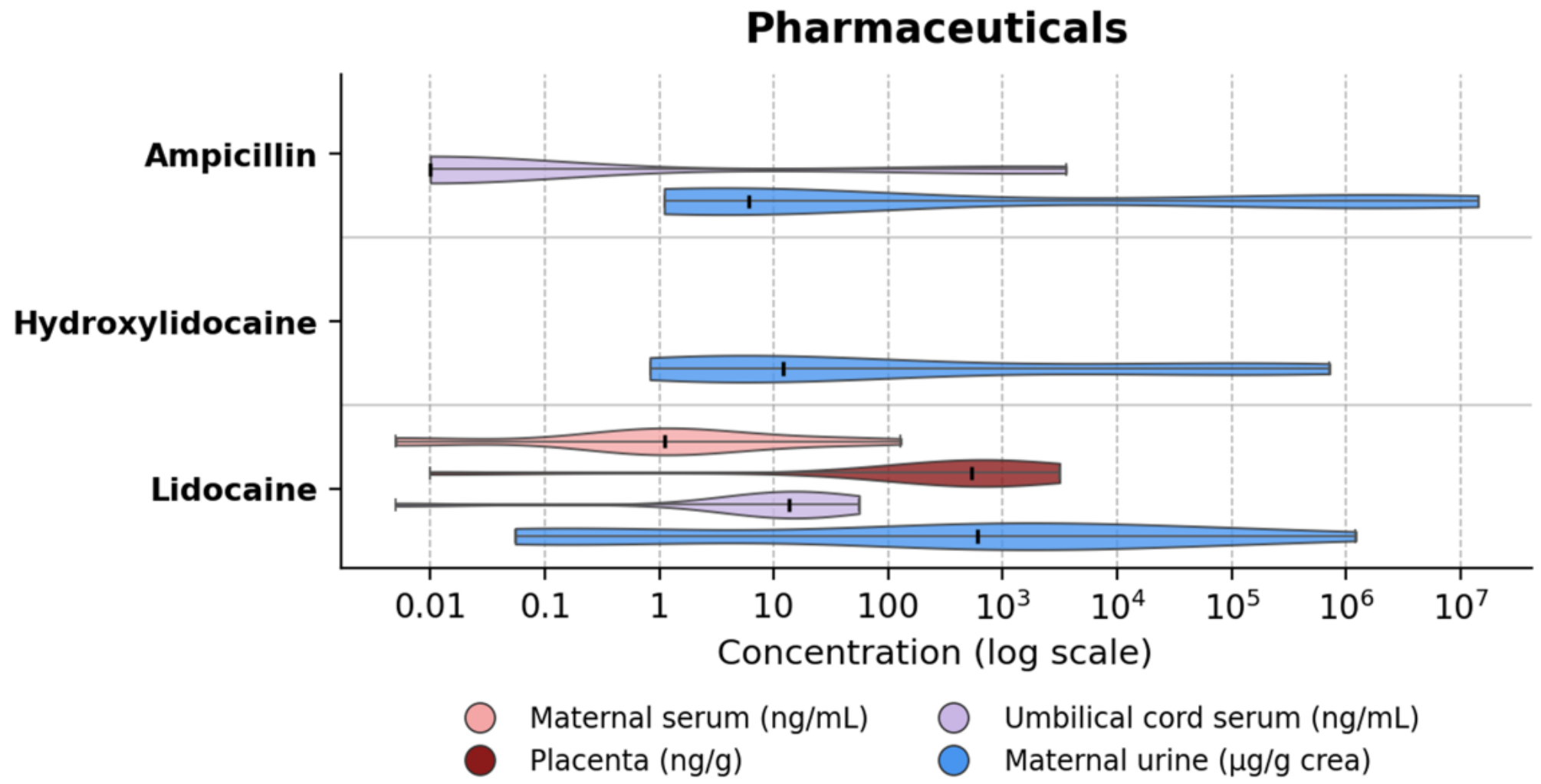

Fig. S4. Violin plots of pharmaceuticals concentrations across biological matrices.
Each distribution represents the log₁₀-transformed concentrations (*ng mL^{-1} for serum, ng g^{-1} for placenta and µg g^{-1} creatinine for urine) of pharmaceuticals in different biological matrices. Violin widths indicate the density of observations, and internal lines represent the 25th, 50th (median), and 75th percentiles. Only chemicals with detection frequencies ≥ 20% in the matrices are shown.

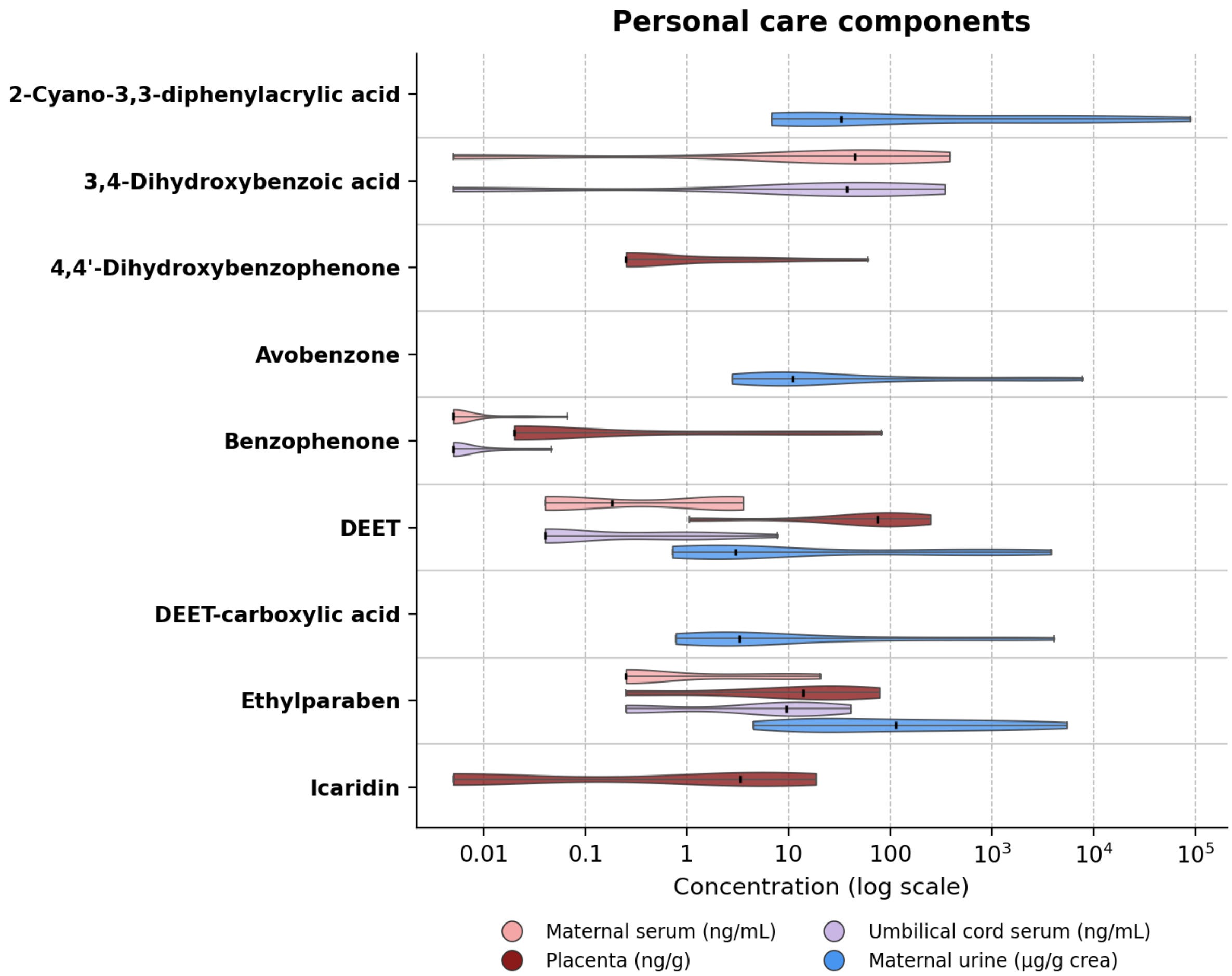

Fig. S5. Violin plots of personal care components concentrations across biological matrices.
Each distribution represents the log₁₀-transformed concentrations (*ng mL^{-1} for serum, ng g^{-1} for placenta and µg g^{-1} creatinine for urine) of personal care components in different biological matrices. Violin widths indicate the density of

observations, and internal lines represent the 25th, 50th (median), and 75th percentiles. Only chemicals with detection frequencies ≥ 20% in the matrices are shown.

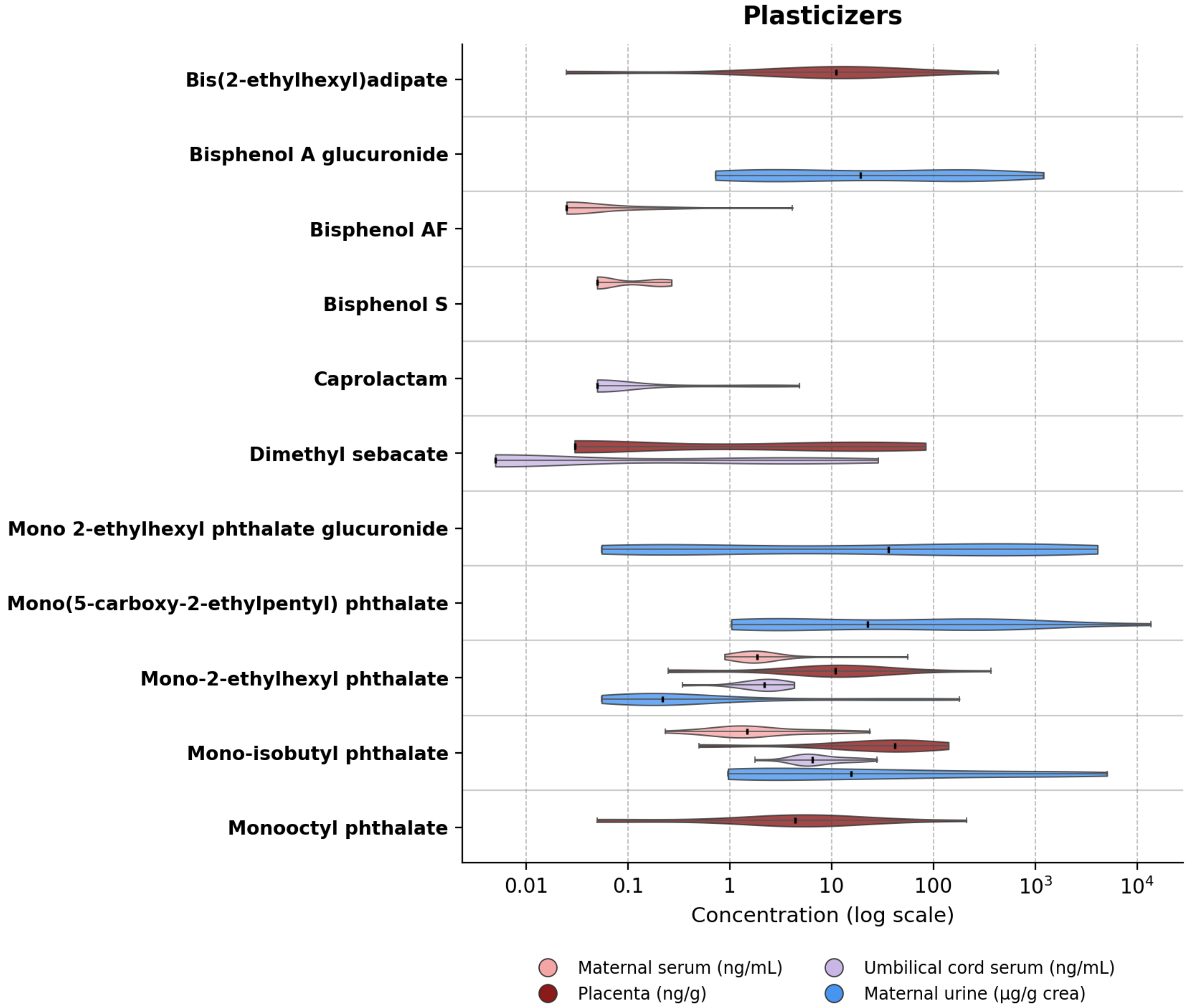

Fig. S6. Violin plots of plasticizers concentrations across biological matrices.
Each distribution represents the $\log_{10}$-transformed concentrations (*ng mL^{-1} for serum, ng g^{-1} for placenta and µg g^{-1} creatinine for urine) of plasticizers in different biological matrices. Violin widths indicate the density of observations, and internal lines represent the 25th, 50th (median), and 75th percentiles. Only chemicals with detection frequencies ≥ 20% in the matrices are shown.

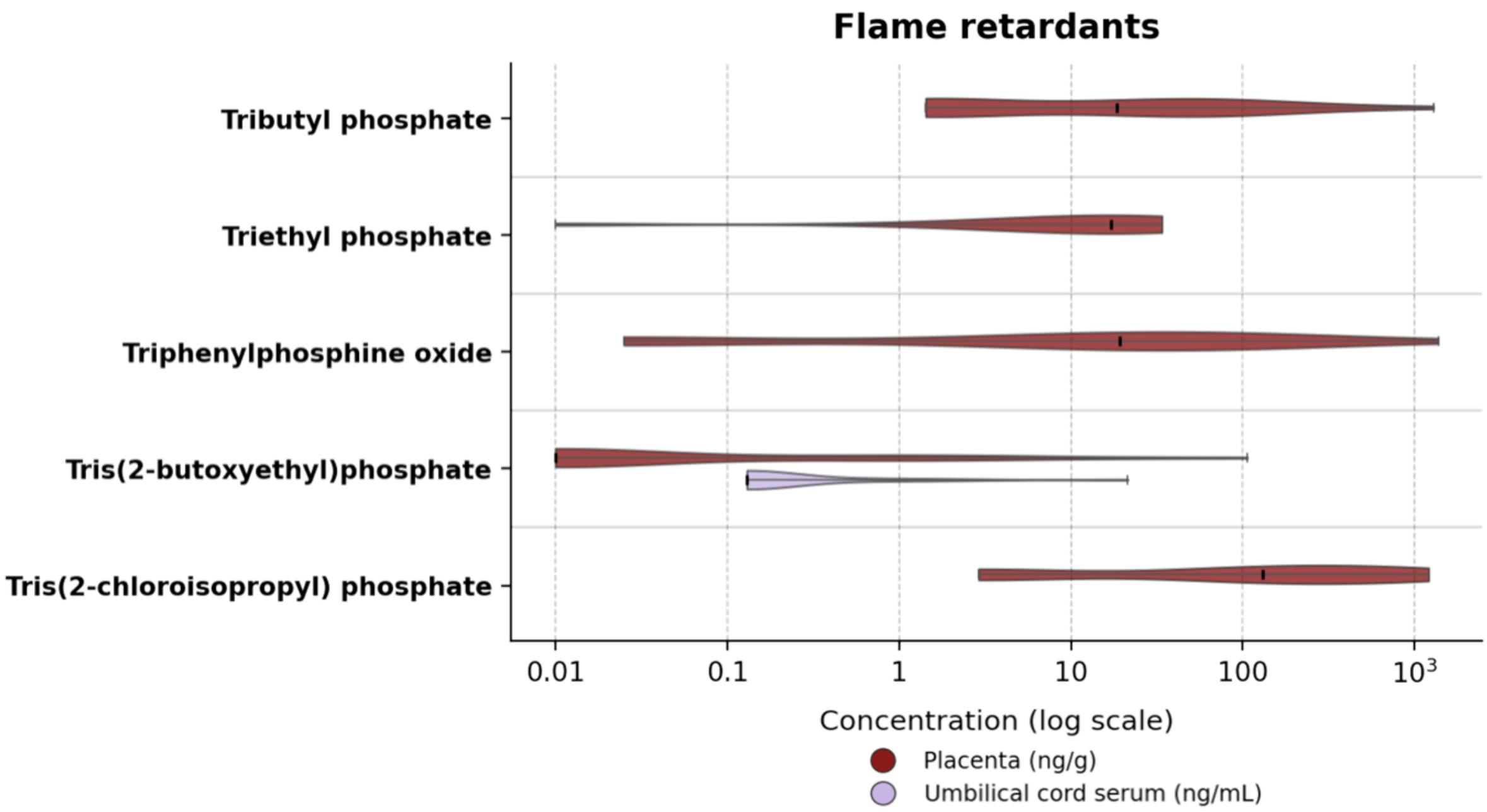

Fig. S7. Violin plots of flame retardants concentrations across biological matrices.
Each distribution represents the log_{10}-transformed concentrations (*ng mL^{-1} for serum, ng g^{-1} for placenta and μg g^{-1} creatinine for urine) of flame retardants in different biological matrices. Violin widths indicate the density of observations, and internal lines represent the 25th, 50th (median), and 75th percentiles. Only chemicals with detection frequencies ≥ 20% in the matrices are shown.

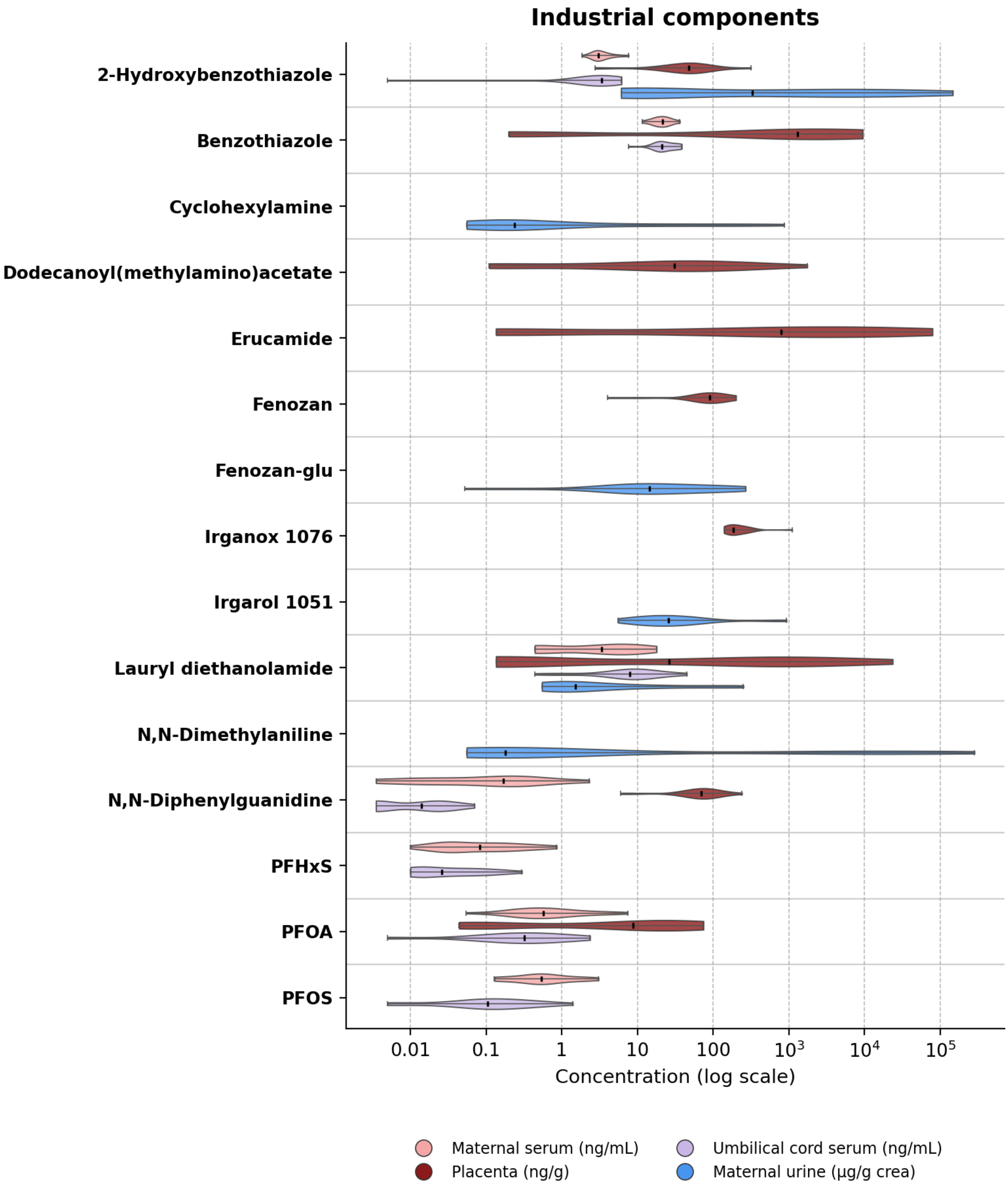

Fig. S8. Violin plots of multipurpose industrial chemicals concentrations across biological matrices. Each distribution represents the $\log_{10}$-transformed concentrations (*ng mL^{-1} for serum, ng g^{-1} for placenta and µg g^{-1} creatinine for urine) of multipurpose industrial chemicals in different biological matrices. Violin widths indicate the density of observations, and internal lines represent the 25th, 50th (median), and 75th percentiles. Only chemicals with detection frequencies ≥ 20% in the matrices are shown.

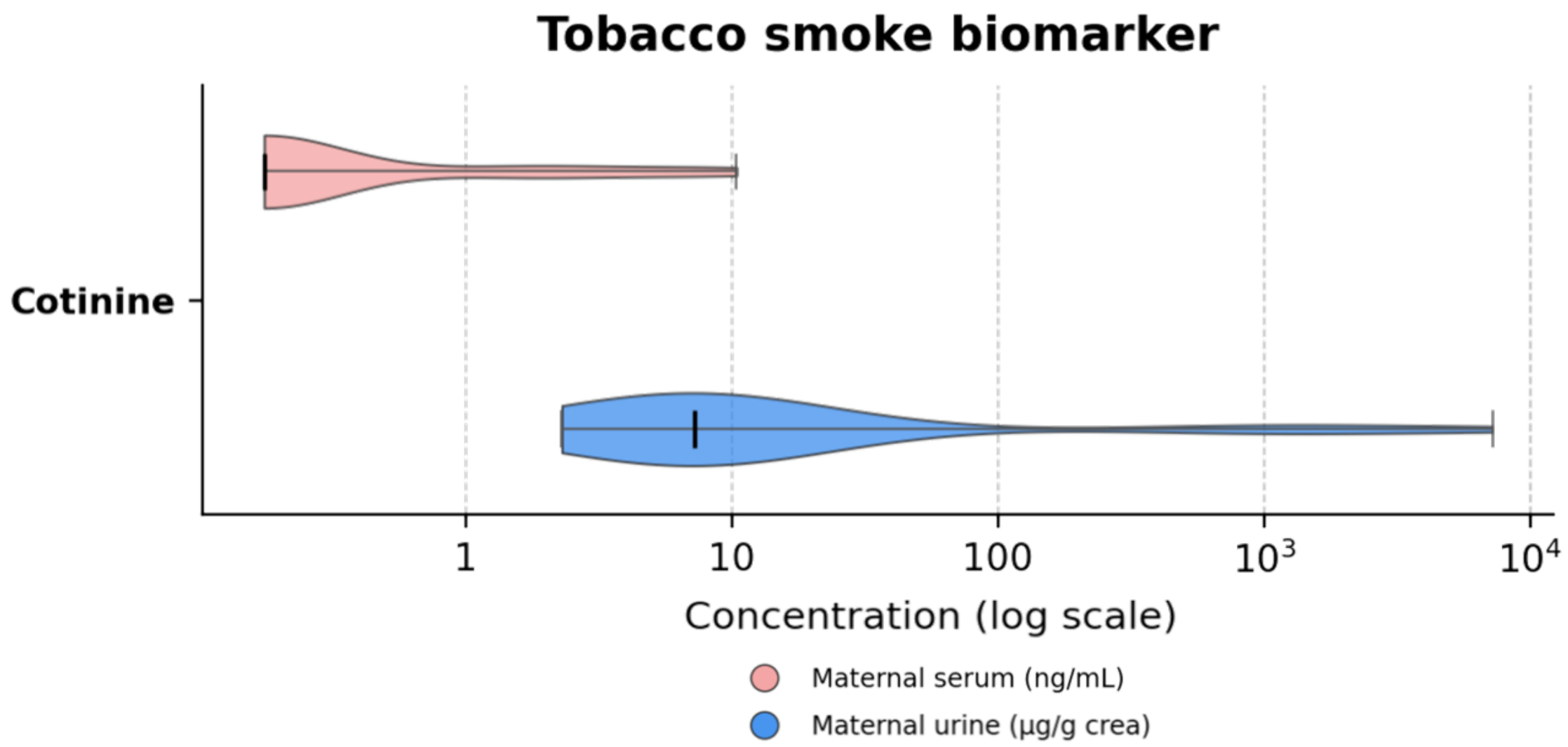

Fig. S9. Violin plots of cotinine concentrations across biological matrices.
Each distribution represents the log_{10}-transformed concentrations (*ng mL^{-1} for serum and µg g^{-1} creatinine for urine for urine) of cotinine in different biological matrices. Violin widths indicate the density of observations, and internal lines represent the 25th, 50th (median), and 75th percentiles. Only chemicals with detection frequencies ≥ 20% in the matrices are shown.

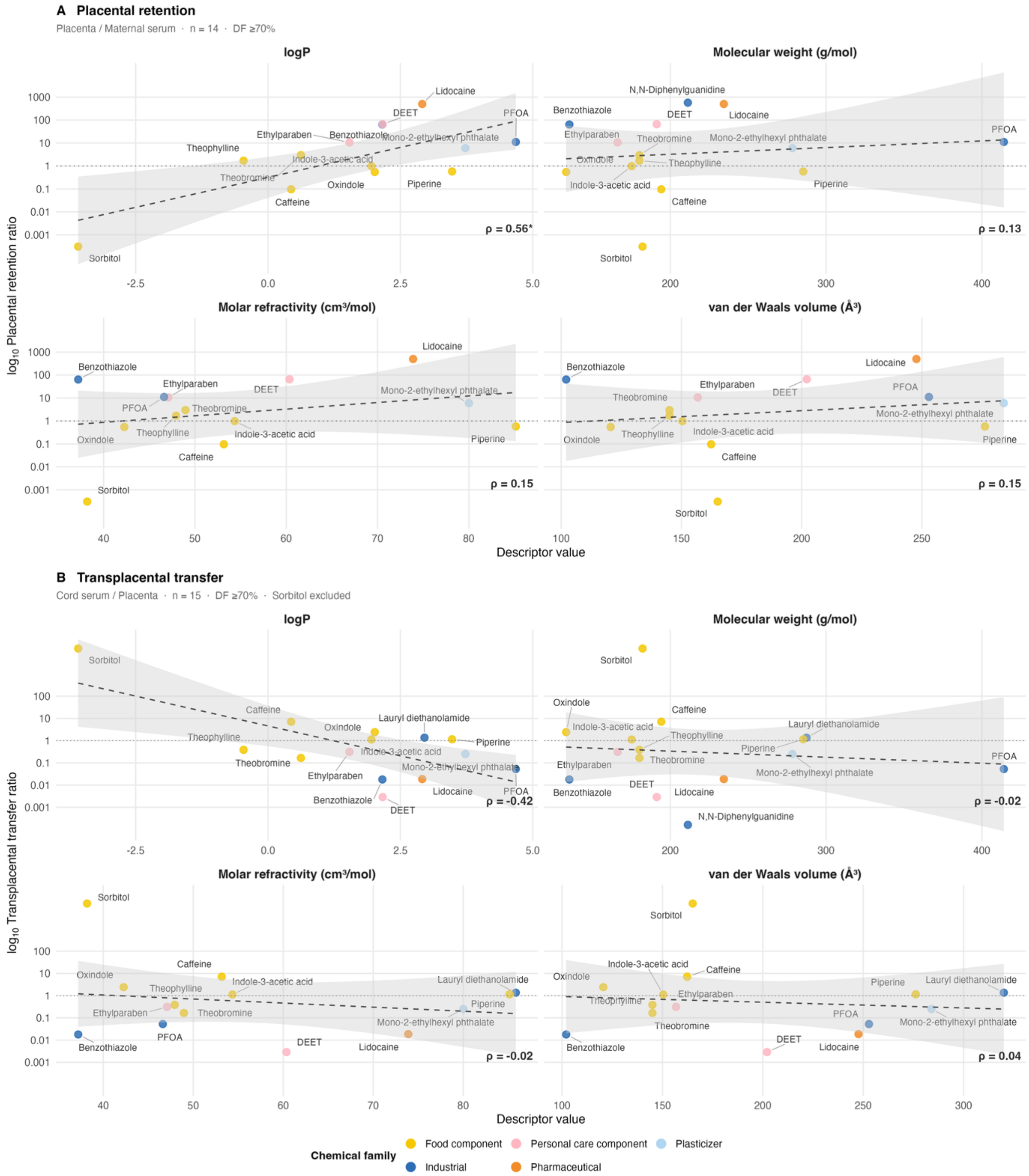

Fig. S10. Relationship between molecular descriptors and placental retention and transplacental transfer ratios.

Scatter plots showing Spearman correlations between four molecular descriptors (logP, molecular weight, molar refractivity, and van der Waals volume) and (A) placental retention ratio (placenta/maternal serum; n = 16) and (B) transplacental transfer ratio (cord serum/placenta; n = 17) for compounds with detection frequency ≥70% in at least one paired matrix (placental retention: maternal serum or placenta; transplacental transfer: placenta or umbilical cord serum). Each point represents one compound, labelled by name and coloured by chemical family. The dashed line represents the linear regression fit with 95% confidence interval (grey shading). The horizontal dotted line indicates a ratio of 1 (equal concentrations in both compartments). Spearman's ρ and significance level are shown in the bottom right corner of each panel (* $p < 0.05$, non-significant correlations shown without asterisk). All ratios are shown on a $\log_{10}$ scale.
